# Word prediction using closely and moderately related verbs in Down syndrome

**DOI:** 10.3389/fpsyg.2022.934826

**Published:** 2022-10-03

**Authors:** Armando Q. Angulo-Chavira, Alejandra M. Castellón-Flores, Julia B. Barrón-Martínez, Natalia Arias-Trejo

**Affiliations:** ^1^Laboratorio de Psicolingüística, Facultad de Psicología, Universidad Nacional Autónoma de México, Mexico City, Mexico; ^2^Facultad de Estudios Superiores Zaragoza, Universidad Nacional Autónoma de México, Mexico City, Mexico

**Keywords:** Down syndrome, prediction, verb restriction, association strength, productive vocabulary

## Abstract

People with Down syndrome (DS) have several difficulties in language learning, and one of the areas most affected is language production. Theoretical frameworks argue that prediction depends on the production system. Yet, people with DS can predict upcoming nouns using semantically related verbs. Possibly, prediction skills in people with DS are driven by their associative mechanism rather than by the prediction mechanism based on the production system. This study explores prediction mechanisms in people with DS and their relationship with production skills. Three groups were evaluated in a preferential-looking task: young adults, children with DS, and a typically developing control group paired by sex and mental age. Participants saw two images, a target and a distractor. They also heard a sentence in one of the three conditions: with a verb that was closely related to the object (e.g., “The woman read the book”), with a verb that was moderately related to the object (e.g., “My uncle waited for the bus”), or with a verb that was unrelated to the object (e.g., “My sister threw a broom”). Their productive vocabulary was then measured. In the young adult and typically developing groups, the results showed prediction in sentences with highly and moderately related verbs. Participants with DS, however, showed prediction skills only in the highly related context. There was no influence of chronological age, mental age, or production on prediction skills. These results indicate that people with DS base prediction mainly on associative mechanisms and they have difficulty in generating top-down predictions.

## Introduction

Lexical prediction allows people to anticipate information based on the top-down pre-activation of potential word candidates (Schoknecht, 2022) and respond rapidly and assertively to linguistic information (Federmeier, 2007; Fine et al., 2013; Kuperberg and Jaeger, 2016). Children can use different kinds of cues to make predictions, including the transitional probability between words (Pelucchi et al., 2009), phonological forms, prosody (Ito and Speer, 2008; DeLong et al., 2015), morphology (Martin and Ellis, 2012; Arias-Trejo et al., 2013; Huettig and Brouwer, 2015), syntaxis (Federmeier and Kutas, 1999), and sentence context (Campanelli et al., 2018).

There is extensive evidence about prediction using semantically related verbs (Altmann and Kamide, 1999; Mani and Huettig, 2012, 2014). Altmann and Kamide (1999) showed that adults anticipate referents based on the semantic attributes of a verb. On hearing *eat*, they looked more at the image of an edible object than a non-edible one. There are also several studies on prediction during language comprehension in younger populations, such as infants and school children with typical development (TD), that demonstrate prediction skills as early as 24 months of age (Borovsky et al., 2012; Mani and Huettig, 2012; Lukyanenko and Fisher, 2016; Mani et al., 2016; Gambi et al., 2018). However, little is known about people with genetic syndromes that lead to different developmental trajectories (Arias-Trejo et al., 2019).

Down syndrome (DS) is a genetic disorder caused by all or part of an extra copy of chromosome 21 (Lubec and Engidawork, 2002). One in every thousand babies born presents DS. It is the most frequent biological cause of intellectual disability (Dierssen, 2012), resulting in cognitive development that falls behind chronological age (van Gameren-Oosterom et al., 2011). One area of disadvantage in children with DS is language production. Part of this disadvantage is related to physical abnormalities in the vocal apparatus (Kumin et al., 1994), including a small oral cavity, irregular teeth, a large tongue, and abnormalities in the facial muscles. They are usually affected by hearing loss and otitis media, which affect not only comprehension but also perception of speech during oral production (Dodd and Thompson, 2001).

Word comprehension scores in children with DS are similar to those of their TD peers matched by mental age (see Næss et al., 2011). Their comprehension of nouns and verbs, for instance, is remarkably well preserved (Michael et al., 2012), but they experience problems with grammar (Witecy and Penke, 2017), use of contextual cues (Hsu, 2019), and syntax (Iverson et al., 2003). In general, word production is lower in children with DS than in those with TD (Roberts et al., 2007; Martin et al., 2013).^1^ They usually present delayed speech (Roberts et al., 2005) and speech errors (Rosin, 1988). In a longitudinal study, Næss (2022) evaluated children with and without DS with similar non-verbal mental age, auditory memory, oral motor skills, and receptive vocabulary and found a slower growth of expressive vocabulary in people with DS than those with TD. People with DS also experience morphosyntactic difficulties in production: problems with gender and number agreement between articles and nouns (Eadie et al., 2002), and errors in grammatical categories, including verbs, in spontaneous speech (Chapman et al., 2000; Chapman, 2006). They also have problems producing grammar, morphemes, and syntax (Yoder et al., 2006), and problems with semantic processing (Laws et al., 2015; Andreou and Katsarou, 2016; cf. Barrón-Martínez and Arias-Trejo, 2020, 2022). Andreou and Katsarou (2016) evaluated the semantic performance of adolescents with DS with a mental age of 3.5–6.5 years, using tests that measured receptive and expressive semantic skills. They found that the group with DS had lower performance on all tests compared to a TD group matched by mental age, and those with DS performed lower on expressive than receptive semantic skills. In sum, the evidence showed generalized language problems in people with DS, and particular weakness in production and semantic processing.

Models of prediction in language comprehension (Dell and Chang, 2014; Pickering and Gambi, 2018) postulate that the production system is highly important to make predictions during comprehension. Experimental evidence has shown that using the production system during language comprehension makes predictive processing difficult; people are not able to make predictions while they produce syllables during comprehension tasks (Martin et al., 2018). Verbal fluency is also related to prediction skills (Rommers et al., 2015). In a correlational study with 2-year-old German toddlers, Mani and Huettig (2012) found a positive correlation between their ability to predict a target object using a semantically related verb and their productive vocabulary: high-scoring producers predicted the target, but low-scoring producers did not. However, prediction skills did not correlate with comprehension scores (Borovsky et al., 2012; Mani et al., 2016). These results suggest the need for a well-developed production system to make predictions during language comprehension.

If people with DS have production problems, they should therefore also have problems with prediction; however, there is evidence for some prediction skills in this population. In a preferential-looking task using an eye-tracker, Arias-Trejo et al. (2019) reported that children with DS (mental age: 5.48 years), as well as their TD peers matched by mental age, used the semantic information contained in a verb (e.g., *eat*) to anticipate an edible target (e.g., *cake*) in preference to a non-edible distractor. Thus, the question is, if people with DS have problems with production, how do they make predictions about upcoming linguistic information? Understanding this predictive processing in people with DS is essential to understanding their language difficulties because children learn a language using prediction and prediction errors (Dell and Chang, 2014; Reuter et al., 2019).

Two mechanisms cooperate to create predictions during language comprehension: prediction-by-association and prediction-by-production (Huettig, 2015; Pickering and Gambi, 2018). Prediction-by-association mechanism is a bottom-up mechanism of automatic spreading activation based on representations shared between words. This mechanism has been described extensively in priming studies (Collins and Loftus, 1975; Dell, 1986; Anderson, 2013); it is essentially predictive since the activation spreads among concepts before the presentation of the target word (Huettig, 2015; Pickering and Gambi, 2018). The word *dog*, for example, pre-activates the word *bone* because these words occur together in speech and the environment. The prediction-by-association mechanism is inefficient because the activation spreads freely through all related^2^ concepts, regardless of the context. For instance, in the sentence “My dog is chasing a cat,” the activation from *dog* can pre-activate *cat* and other incongruent but related words like *bone*. Nevertheless, the cognitive load is low, and the pre-activation is virtually instantaneous. The prediction-by-production mechanism is more efficient because it considers contextual information, both linguistic and non-linguistic, to make predictions, but these predictions are slower and require more cognitive processing. Pickering and Gambi (2018) argue that the top-down predictions generated by this second mechanism are based on the production system: to make predictions during the comprehension process, the production system predicts the concept of the word based on linguistic and non-linguistic context. Notably, these two mechanisms, prediction-by-production and prediction-by-association are complementary: the extent to which predictions rely on one system or the other depends on the availability of information, resources, and time (Pickering and Gambi, 2018).

According to prediction theory, the production problems of people with DS should result in difficulties in creating top-down predictions using contextual information, but not bottom-up automatic predictions. Recent studies have shown that children with DS may use pre-activation mechanisms based on the association between concepts (Barrón-Martínez and Arias-Trejo, 2020, 2022). Barrón-Martínez and Arias-Trejo (2022) evaluated children with and without DS in a preferential-looking task using an eye-tracker. In half of the trials, participants were exposed to pairs of words (prime and target) that were related, and in the other half to pairs that were unrelated. The participants looked more at a named target image preceded by a related prime than one preceded by an unrelated prime. This finding suggests that the prediction-by-association mechanism is preserved in children with DS.

Arias-Trejo et al. (2019) demonstrate that children with DS can use the verb information to make predictions. Verbs provide information about the action and important semantic and grammatical information about the agent and patient of the action. These thematic roles are verb-specific concepts (McRae et al., 1997; Ferretti et al., 2001). For example, the verb *gamble* activates information about the location of the action (e.g., *casino*) and possible participants in the action (e.g., *gambler*).

The information provided by verbs can be used in making predictions (Altmann and Kamide, 1999); however, these predictions are not tied to the verb itself but to the event in which verbs occur together with agents and patients. Kamide et al. (2003) evaluated young adults using the visual world paradigm. Participants were presented with an array of images, including several objects: a motorcycle, a carousel, a man, and a girl. When participants heard the sentence “The man will ride the…,” they looked at the motorcycle, but not the carousel; when they heard the sentence “The girl will ride…,” they looked at the carousel. Thus, although verbs can be linked to specific noun concepts, the elicited link depends on the context.

Stefaniak et al. (2021) found developmental differences using contextual information. In Experiment 2, school-age children and adults performed a grammatical judgment task, including both typical and unusual (but grammatically correct) patients for verbs. They found that both groups showed better performance with typical than with unusual patients; however, younger children showed lower performance in judging unusual patients. The authors interpreted these results based on the declarative/procedural model: in the processing of typical patients, the declarative memory assigns a meaning, and the procedural memory evaluates whether the patient can be used with the verb; with unusual patients, the declarative memory does not generate meaning, and the procedural memory does not evaluate the patient.

Stefaniak et al. (2021) argue that spreading activation has little influence on the verb-patient typicality effect since there was no variation from the free association norms, and the task relied more on the syntactic cues and the thematic roles. This interpretation is congruent with our theoretical framework: the prediction-by-association system always generates predictions, and the prediction-by-production system uses contextual information such as syntax or grammar to generate predictions requiring more information. For example, the verb *read* is highly associated with the patient noun *book*; in this case, the prediction relies more on the prediction-by-association system. However, the lower degree of association between the verb *wash* and the patient *bucket* relies less on prediction-by-association and more on prediction-by-production because the verb *wash* can be applied to different objects. In the latter case, additional contextual information is needed to formulate a correct prediction.

The present study examines whether children with DS anticipate a referent in the same way as their mental age-matched peers in two different contexts: when there are higher and lower levels of association between verbal cues. We hypothesized that in a predictive sentence with a high degree of association between the verb and the target noun (e.g., *read*—*book*), children with and without DS would look at the target image before it was named because they would rely on prediction-by-association. However, in a sentence with a lower degree of association between the verb and the noun (e.g., *wash*—*bucket*), participants with TD, but not those with DS, would look at the target image before it was named. Here, they need to rely more on the prediction-by-production mechanism; thus, problems with production in people with DS would affect this mechanism. We also hypothesized that vocabulary production would modulate prediction in DS, as participants with higher vocabulary production scores would use their greater production skills in predictive sentences with low associations between the verb and the target noun.

## Materials and methods

### Participants

The study was carried out online because of the COVID-19 pandemic. We evaluated 21 participants with DS with a mean chronological age of 20.784 years (*SD* = 5.754, range: 11.460–29.563) and a mean verbal mental age of 5.524 years (*SD* = 2.363, range: 3.5–13.83). Five were non-verbal and therefore did not produce any language. All participants with DS lived in a monolingual environment, according to their parents or primary guardian. We also evaluated a control group of children with TD paired by mental age and sex with the participants with DS (see Table 1). This group included 21 participants with a mean chronological age of 5.524 years (*SD* = 2.363, range: 3.25–13.58) and a mean verbal mental age of 5.829 years (*SD* = 2.418, range: 3.25–13.83). Another 21 participants with TD were excluded because they had a mental age greater than their chronological age and could not be paired with participants with DS. All were monolingual Spanish speakers. According to parental reports, all participants had a normal or corrected-to-normal hearing and vision and had no neurological/psychiatric problems. An additional group of 39 adults was assessed (*M* = 23.87 years, *SD* = 2.48, range: 18–28, 22 male) to test the functioning of our experimental manipulation. Three adults were excluded from this group because of failures in calibration. All participants, or, in the case of minors, their parents or guardian, provided informed consent. The study was approved by the research ethics committee of the Facultad de Psicología, Universidad Nacional Autónoma de México (Approval No. FCPE_13092021_H_AC).

**TABLE 1:** Socio-demographic data.

		TD	DS	*P*-value
Age	M (SD)	5.524 (2.363)	20.936 (5.765)	<0.001
Sex	N (male/female)	11/10	11/10	–
Mental age	M (SD)	5.829 (2.418)	5.773 (2.482)	0.941
Productive vocabulary	M (SD)	51.904 (22.248)	43.600 (27.400)	0.185

*P*-value corresponds to an independent sample test between the two groups. TD, typical development; DS, Down syndrome.

### Instruments

#### Mental age: Receptive vocabulary assessment

Participants’ verbal mental age was evaluated to match participants from the TD and DS groups and to determine whether cognitive development affected linguistic prediction skills. Verbal mental age was measured with remote administration of the Receptive One-Word Picture Vocabulary Test: SBE (Martin, 2010b). Participants were presented with four images and asked to match a word they heard to the correct image. The test was suspended after four consecutive errors or failure to respond to six stimuli. The raw score was calculated by subtracting the number of errors from the total number of items reached and converted to mental age using standardized tables (Martin, 2010b). The approximate duration of the test was 20 min. For younger children and participants with DS who had difficulty verbally indicating the image, parents or guardians were asked to indicate the images the child had pointed to, even if they were incorrect. The test administrator corroborated the answers by noting the part of the screen the participant pointed to. We used this mental age evaluation because our experimental task measures prediction during language comprehension; it is thus an appropriate measure for pairing participants with similar comprehension skills since receptive vocabulary is a good predictor of general comprehension (Ricketts et al., 2007; Stolt et al., 2016; Cheung et al., 2022). This evaluation also has two methodological advantages: there are normative values for the Mexican Spanish-speaking population and it can be performed online.

#### Productive vocabulary assessment

Participants’ expressive vocabulary was evaluated to determine the effect of production skills on language prediction. The Expressive One-Word Picture Vocabulary Test: SBE (Martin, 2010a) was administered remotely. Participants were presented with one image and asked to name it. The test was suspended after four consecutive errors. In this evaluation, some participants with DS scored zero points; they were non-verbal according to their parents. However, we assumed that participants understood the task because they followed the instructions for the mental age evaluation and the experimental task. The raw score was calculated by subtracting the number of errors from the total number of items reached. The test was suspended if participants failed to respond to six stimuli (Martin, 2010a). The approximate duration of the test was 20 min. Parents were asked to avoid interaction with participants while they performed the test.

### Materials

Three types of sentences were created: predictable sentences with a closely related verb (CV; e.g., “The woman read the book”), a moderately related verb (MV; e.g., “My uncle waited for the bus”), and unpredictable sentences with an unrelated verb (UV; e.g., “The woman lost the sock”). A total of 56 sentences were created, 14 for the CV condition, 14 for the MV condition, and 28 for the UV condition. All words used in the sentences were familiar to children (Alva-Canto, 2001). Verbs and direct objects in the CV condition had a high association strength and those in the MV condition had a lower association strength, according to the validation studies described below. The UV sentences used the same target nouns as the predictive sentences but with unrelated verbs. Supplementary Appendix 1 shows the experimental sentences, the targets, and distractors in the CV condition, and their corresponding UV sentences. Supplementary Appendix 2 shows the experimental sentences, the targets, and distractors in the MV condition, and their corresponding UV sentences.

The sentences were audio recorded in a female, child-directed voice, with no specific emphasis on any part of the sentence, in a quiet room (a basement with low noise levels), using a Shure MV51 microphone at 44,100 Hz and 16-bits. They were edited in Adobe Audition CS6 with noise reduction, normalization, and sound amplification. The lists of sentences were recorded four times in different orders. First, they were recorded in ascending order (from the beginning to the end of the list) and then in descending order, and then the sequence was repeated.

Two objects were presented visually as competitors: a target and a distractor (Supplementary Table 1). The target was the noun that appropriately completed the sentence for the closely and moderately related grammatical constructions. The UV condition used the same target and distractor as the CV and MV conditions. The visual stimuli were realistic photographs of the targets and distractors. The images were edited in Adobe Photoshop CS6 and adjusted to 600 × 600 pixels. Individual images were placed on a gray background (RGB: 225, 225, 225; 1920 × 1080 pixels). The visual and auditory stimuli were then embedded in AVI videos created with Adobe Flash CS6 and Adobe Premiere Pro.

### Sentence validation studies

Two pilot studies were carried out to determine the plausibility of each sentence and the degree of association between the verb and the expected noun. The first evaluated whether the sentences would be likely to be heard in an everyday context (Supplementary Appendix 3). Thirty undergraduates (*M*_*age*_ = 25.3, 17 male) evaluated the plausibility of the sentence with the target (e.g., “The woman read the book”) and with the distractor (e.g., “The woman read the sock”). Kruskal–Wallis tests found differences between conditions in the target (*X*^2^ = 34.707, *p* < 0.001) and the distractor (*X*^2^ = 24.996, *p* < 0.001). *Post-hoc* analysis with a Mann–Whitney *U*-test showed that the CV sentences have more plausibility with the target (*z* = 2.987, *p* = 0.002) but less plausibility with the distractor (*z* = 4.412, *p* < 0.001) than the MV sentences. They also have more plausibility with the target (*z* = 4.896, *p* < 0.001) but less plausibility with the distractor (*z* = 4.483, *p* < 0.001) than the UV sentences. The MV sentences have more plausibility with the target (*z* = 4.243, *p* < 0.001) than the UV sentences; however, there are no differences between MV and UV sentences with the distractor (*z* = 0.480, *p* = 0.644). These results confirm that our predictable sentences are considered more natural than the non-predictable ones, which is expected because regularity generates prediction in language.

The second validation was an association strength task. A “restricted” association task was performed to determine the association levels between the verbs and the nouns in the sentences. A total of 30 university students from Mexico City participated (*M*_*age*_ = 26.2, range: 18–30; 19 male). The pilot experiment was created on the Cognition platform (Cognition Run, 2021), and it lasted approximately 10 min. Participants were asked to write a verb in response to the noun stimulus in this task. The instructions to the participants were: “Next, you will see a series of nouns; please write the first VERB that comes into your mind when reading the noun. Answer as quickly as you can.” Table 2 shows the association strength of the experimental stimuli.

**TABLE 2:** Association strength between targets and distractors.

ID	CV		UV		ID	MV		UV	
	Target	Distractor	Target	Distractor		Target	Distractor	Target	Distractor
1	80	0	0	0	15	23.33	0	0	0
2	56.66	0	0	0	16	16.66	0	0	0
3	70	0	0	6.66	17	20	0	0	0
4	73.33	0	0	6.66	18	6.66	0	0	0
5	60	0	0	0	19	13.33	0	0	13.33
6	66.66	0	0	3.33	20	23.33	0	0	6.66
7	66.66	0	0	0	21	16.66	0	0	0
8	46.66	0	0	0	22	10	0	16.66	0
9	63.33	0	0	0	23	20	6.66	0	6.66
10	53.33	0	0	0	24	20	0	0	0
11	53.33	0	0	0	25	6.66	0	0	0
12	46.66	0	0	0	26	13.33	3.33	0	0
13	73.33	0	0	0	27	20	0	0	0
14	56.66	0	0	0	28	3.33	0	0	0

The ID corresponds to the sentences presented in Supplementary Appendices 2 and 3. CV, closely related verb; MV, moderately related verb; UV, unrelated verb.

A Kruskal–Wallis test revealed significant differences between conditions in the association strength between the verb and the target (*X*^2^ = 49.353, *p* < 0.001) but not between the verb and the distractor (*X*^2^ = 3.526, *p* = 0.172). The exploration of significance analysis showed that the CV condition had a greater association strength between the verb and the noun than the MV (*z* = 4.491, *p* < 0.001) and the UV (*z* = 6.103, *p* < 0.001); the association was greater in the MV than the UV (*z* = 5.555, *p* < 0.001). These results corroborate changes in the association strength between conditions.

Finally, we found a strong positive correlation between the association strength and the plausibility values (*r* = 0.639, *p* < 0.001), implying a relationship between the verbal association and sentence plausibility.

An additional validation study was performed after the review process. This validation study was not used in the stimulus selection, but it is important to corroborate the association strength in both directions between the verb and the expected noun. This additional validation used the same procedure as the original verb association validation, except that the verb was presented as a cue, and participants were asked to provide the first word that came to mind when they saw it. We compared only the CV and MV conditions because all the values in the UV condition were zero. A Mann–Whitney *U*-test showed that the CV condition had higher association values than the MV condition (*Z* = 3.37, *p* < 0.001). The values for the MV condition were very close to zero (*M* = 3.225; *SD* = 5.93), suggesting that the verb does not elicit the expected noun (see Supplementary Appendix 4), and participants probably need the visual context to create a prediction.

### Procedure

The participants were recruited through informational posts on social media and specialized care foundations for people with DS. Parents who contacted us were told about the procedures and objectives of the study and then formalized their participation by signing the informed consent. A socio-demographic questionnaire was first administered to participants’ parents on a Zoom video call to verify that they met the inclusion criteria.

The gaze of the participants was recorded remotely using the RealEye.io online platform. This platform is a webcam-based eye-tracker with a maximum sample rate of 60 Hz; it calculates the gaze position when participants look at their personal computers with an accuracy of approximately 100 px (∼1.5 cm) and with a visual angle error of ∼ 4.17 degrees (RealEye, 2020). This accuracy is appropriate for a two-image visual display and fixation analysis.

Two calibration processes were performed with RealEye. Participants first tracked points using the computer mouse and then performed standard calibrations in which they looked at four different points on the screen. The platform does not store the participant’s image, sound, or location data, but only their gaze position.

**FIGURE 1:**
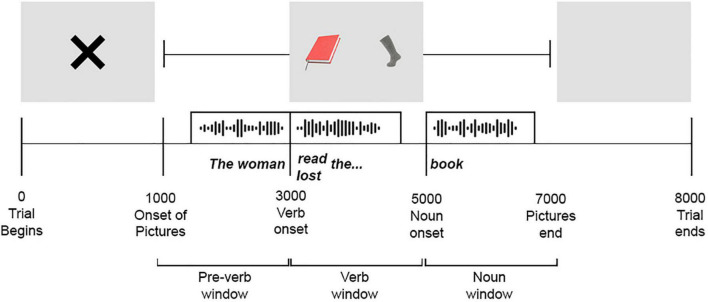
Example of an experimental trial. The image used a creative commons zero (cc0) license and it is the image of public domain.

Each participant heard 28 sentences: 7 CV sentences, 7 MV sentences, and 14 UV sentences. The sentences were counterbalanced across subjects in four different orders so that each pair of images was presented only once to each participant. Each trial had a duration of 8,000 ms. From 0 to 1,000 ms, a fixation point was presented on the screen. The images of the two competitors were presented from 1,000 to 7,000 ms (see Figure 1). The sentence (e.g., “The woman read the book”) was presented as follows: the subject (e.g., “The woman”) was presented in a pre-verb window from 1,000 to 3,000 ms and the verb from 3,000 to 5,000 ms. The verb (e.g., “read”) was presented at 3,000 ms, followed by the determiner (e.g., “the”) and then a period of silence. Then, in the noun window, the direct object (e.g., “book”) was presented from 5,000 to 7,000 ms. Finally, the screen was blank from 7,000 to 8,000 ms.^3^

The receptive and expressive tests were administered in a Zoom video call with support from the participant’s parent or guardian to manage the practical details. The total duration of the evaluations was approximately 90 min. The results of the scales were delivered to the parents in a report that also contained suggestions for educational intervention.

### Data processing

As noted earlier, the data quality of the webcam eye-tracker is lower than that usually employed in an experimental laboratory, but enough for our experimental design. The raw fixation signal was thus interpolated and filtered to enhance robustness and precision (Wass et al., 2014). A Gaussian filter (σ = 5) was applied to reduce high-frequency noise and enhance the precision of the data. A linear interpolation was applied to reconstruct the missing data and standardize the sample rate across participants. To obtain a better reconstruction, signal segments with >150 ms of missing data were not interpolated (Wass et al., 2014). We also adjusted all signals to a sample period of 20 ms (50 Hz); the maximum sample period of the webcam eye-tracker is ∼16 ms (60 Hz). This standardization of the sample rate allowed us to compare changes in temporality between groups and conditions; otherwise, comparing the average looking time over the trial could produce type II errors. Since we hypothesize that participants with DS had weaker prediction skills, avoiding this type of error is important.

Since participants performed the experiments on their computers, there was variation in the size and location of competitors on screens. We thus adjusted the areas of interest by modifying them in proportion to the screen size. The original areas of interest measured 960 × 1,080 pixels and were embedded on a 1,920 × 1,080 background. If, for example, the participant’s screen measured 1,600 × 1,200 pixels, the areas of interest should measure 800 × 1,200 pixels. Changes in height and width were independent to enhance the adjustment. A similar process was applied to the location of the areas of interest. The original location of the upper–left competitor was at 480 × 540 pixels; in the same example, the new location would be at 400 × 600 pixels.

The fixations on the two areas of interest were coded as 1 when the gaze signal coordinates were located inside the area of interest; otherwise, they were coded as 0. Each trial thus had two binary time series indicating when participants looked at any specific competitor. Since participants could only fixate on one competitor at a time, an increased fixation on one competitor implies a decreased fixation on other competitors. To reduce the autocorrelation (temporal dependence between samples) of the fixation signals, we binned the data by averaging it every 100 ms (Mirman, 2014).

Trials were excluded in which participants looked <25% of the time when the competing pictures were present (0–6,000 ms, relative to the picture presentation).

### Statistical analysis

Growth curve analysis (Barr, 2008; Mirman, 2014) and a cluster-based non-parametric test (Maris and Oostenveld, 2007) were used to analyze the prediction over the time course of the trial. The growth curve analysis compared the temporal dynamic among conditions and groups from the verb presentation until the end of the picture presentation (4,000–6,000 ms relative to the picture presentation). This analysis window allowed for modeling the predictive and non-predictive responses using low-order polynomials. The analysis was performed in R version 4.1.1 (R Core Team, 2019) using the glmmPQL function of the mass package. We used a mixed-effects binomial logistic regression because the fixations are binary variables (fixated or not). The dependent variable was the log odds ratio of the fixation computed as follows (Barr, 2008): $l\,o\,g\,\frac{F}{N-F}$, where *F* is the sum of fixations in a specific bin and *N* is the total number of fixations in the bin. The time was modeled using third-order orthogonal polynomials (Mirman, 2014). The fixed effects of the model were all-time terms (linear, quadratic, and cubic), condition (CV, MV, or UV), and group (TD or DS). For the random effect, we used the maximal random structure that allowed convergence (Barr, 2008); for all analyses, the maximal random structure was the slope of all time terms on the subject and the intercept of the trials. The categorical variables were dummy coded using the UV condition and the TD group as a reference.

The cluster-based non-parametric test better describes the temporality of prediction effects (beginning, duration, and end); these were evaluated from the onset to the end of the picture presentation (0–6,000 ms). To compare conditions, we used paired *t*-tests contrasting CV and MV conditions against the UV condition, independently for each group. We also compared each condition against chance level (0.5) using a one-sample *t*-test; this comparison was performed independently for each group and condition. Clusters were created by summing the adjacent *t*-values higher than the critical value for α = 0.05 (adults: 2.02; TD and DS groups: 2.08). The permuted distribution (100,000 iterations) was created by shuffling the data randomly between conditions for paired tests and shuffling the mathematical sign for the one-sample test. In each iteration, we took only the maximum permuted cluster. A cluster was significant if its value was less than 5% of the total values of the permuted distribution.

Using the model comparison approach, we also evaluated the effect of chronological age, mental age, production, and association strength on prediction in the DS group. Chronological age was used to assess the influence of language experience, mental age was used to evaluate the effect of cognitive development, and production was used to determine the effect of preservation of the productive system on prediction skills. The association strength between the verb and the expected noun was used to evaluate whether the participants with DS had better predictions when there was a high degree of association. The fixation data were aggregated from 2,500 to 4,000 ms, relative to the picture presentation: the period in which participants could predict the upcoming noun. All continuous variables were min-max normalized (−0.5 to 0.5) to improve the convergence of the model. The categorical variables were dummy coded using the UV condition as a reference. Binomial mixed effect models (the glmer function) were compared using the change in log-likelihood (−2 times) with a chi-squared distribution. Thus, we first created a reference model including only the condition as fixed effects and the subjects on the slope of the condition, and the intercept of the items as a random effect. The demographic variables were then included independently in the reference model. We also computed the Bayes factor using the package bayestestR (Makowski et al., 2019) to provide evidence for the null or alternative hypothesis. If the Bayes factor was <0.33 (Wetzels et al., 2011), we assumed that the variable was not relevant to the explanation of the predictive effect.

## Results

### Adults

**FIGURE 2:**
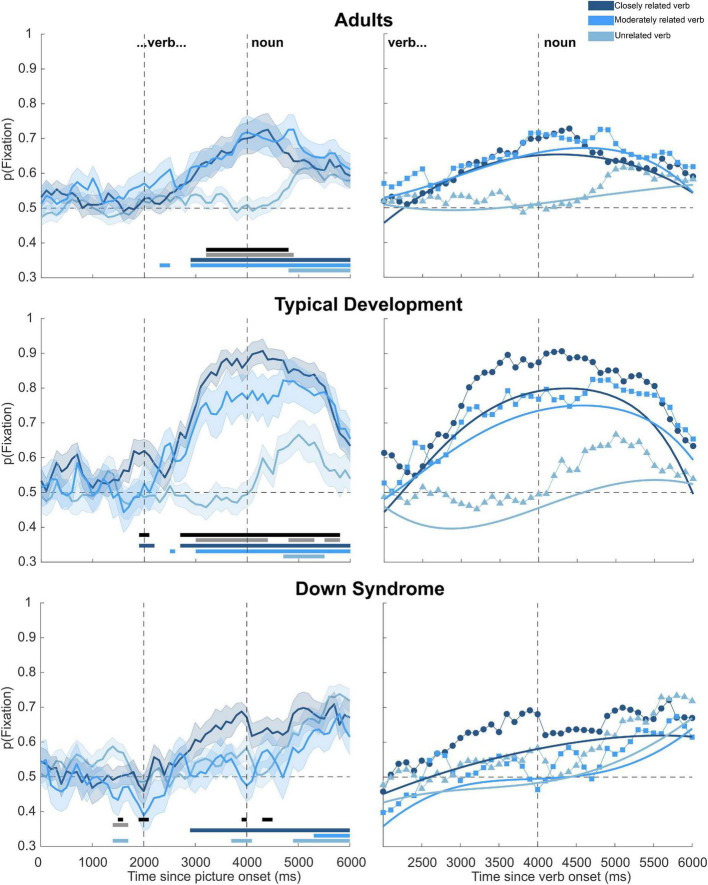
Probability of fixation and fitted lines for all groups. Left panels lines represent the average probability of fixation. Shaded areas show standard error. The horizontal dashed line indicates chance level; the vertical dashed lines the presentation of the verb and the noun. Horizontal bars in the lower part of each plot indicate the significant clusters. Closely related and unrelated verb differences are shown in black, and moderately related and unrelated verb differences are shown in gray. Blue lines represent the difference with chance level (0.5), and the colored lines correspond to the colors of the conditions. Right panels lines with markers show the average probability of fixation. Solid lines indicate the fitted line from the growth curve analysis. The horizontal dashed line shows the chance level, and the vertical dashed line the presentation of the noun. Note that the time shown in this plot begins with the presentation of the verb. The image used a creative commons zero (cc0) license and it is the image of public domain.

All trials were analyzed for 39 adults (see section “Data processing”); however, three adults were excluded from the final sample because of calibration problems. The upper panels of Figure 2 show the probability of fixation in each condition (left) and the modeled data (right). Preliminary examination revealed an increase in fixation in the CV and MV conditions after the verb presentation and the UV condition after the presentation of the noun.

Table 3 presents the statistical values of the growth curve analysis. The results were significant for the CV and MV conditions, indicating that participants looked more in these conditions than in the UV condition.

**TABLE 3:** Growth curve analysis for adults.

Fixed effects	β	*SE*	*df*	*t*	*p*
Intercept	0.0856	0.107	43591	0.793	0.427
**Linear**	**0.643**	**0.266**	**43591**	**2.414**	**0.015**
Quadratic	0.457	0.294	43591	1.555	0.119
Cubic	−0.104	0.172	43591	−0.603	0.546
**CV**	**0.441**	**0.131**	**1049**	**3.371**	**<0.001**
**MV**	**0.588**	**0.131**	**1049**	**4.474**	**<0.001**
Linear: CV	0.299	0.162	43591	1.849	0.064
Linear: MV	0.274	0.162	43591	1.684	0.092
**Quadratic: CV**	−**2.259**	**0.164**	**43591**	−**13.725**	**<0.001**
**Quadratic: MV**	−**1.898**	**0.164**	**43591**	−**11.516**	**<0.001**
Cubic: CV	0.128	0.163	43591	0.791	0.428
**Cubic: MV**	−**0.490**	**0.163**	**43591**	−**2.990**	**0.002**

Formula: log odds (fixations) ∼ (Linear + Quadratic + Cubic) × Condition + [(Linear + Quadratic + Cubic)| Subject] + (1| Item). Conditions: Unrelated verbs (UV), closely related verbs (CV), moderately related verbs (MV). *SE*, standard error; *df*, degrees of freedom. Bold values indicate significant effects.

According to the interaction of both predictive conditions with the quadratic term, participants had a sharper fixation pattern in both predictive conditions (CV and MV) than in the non-predictive one (UV). Finally, the interaction of the MV condition with the cubic term suggests that participants looked more at the target and looked away faster in the MV than in the UV condition.

The cluster-based permutation analysis revealed that participants looked more in the CV than in the UV condition from 3,200 to 4,800 ms (*t*_*cluster*_ = 53.615, *t*_*max*_ = 4.109, *p* < 0.001), and more in the MV than in the UV condition from 3,200 to 3,900 ms (*t*_*cluster*_ = 59.380, *t*_*max*_ = 4.240, *p* < 0.001). They also looked more at the target than chance level in the CV condition from 2,000 to 6,000 ms (*t*_*cluster*_ = 117.235, *t*_*max*_ = 4.808, *p* < 0.001). Adults looked more at the target than chance level in the MV condition in two time clusters: from 2,300 to 2,500 ms (*t*_*cluster*_ = 7.737, *t*_*max*_ = 3.144, *p* = 0.019) and from 2,900 to 6,000 ms (*t*_*cluster*_ = 125.750, *t*_*max*_ = 5.237, *p* < 0.001). Finally, they looked more at the target than chance level in the UV condition from 4,800 to 6,000 ms (*t*_*cluster*_ = 48.407, *t*_*max*_ = 4.418, *p* < 0.001).

### Typical development and Down syndrome groups

Approximately 95% of the trials (559 of 588) with the TD group and 97% (573 of 588) with the DS group were analyzed. No participants were excluded for missing data or calibration problems in both youngest groups. Table 4 presents the statistical values of the growth curve analysis.

**TABLE 4:** Growth curve analysis for DS and TD groups.

Fixed effects	β	*SE*	*df*	*t*	*P*
Intercept	−0.249	0.179	45262	−1.392	0.163
**Linear**	**1.524**	**0.46**	**45262**	**3.306**	**<0.001**
Quadratic	0.600	0.368	45262	1.628	0.103
**Cubic**	−**0.580**	**0.233**	**45262**	−**2.487**	**0.012**
**CV**	**1.468**	**0.142**	**1086**	**10.304**	**<0.001**
**MV**	**1.326**	**0.145**	**1086**	**9.109**	**<0.001**
Group	0.301	0.251	40	1.197	0.238
Linear: CV	0.204	0.234	45262	0.873	0.382
Linear: MV	0.625	0.245	45262	2.543	0.011
**Quadratic: CV**	−**4.844**	**0.25**	**45262**	−**19.354**	**<0.001**
**Quadratic: MV**	−**3.255**	**0.256**	**45262**	−**12.715**	**<0.001**
Cubic: CV	−0.287	0.244	45262	−1.174	0.240
Cubic: MV	−0.094	0.253	45262	−0.373	0.709
Linear: Group	0.583	0.65	45262	0.897	0.369
Quadratic: Group	0.266	0.518	45262	0.514	0.607
**Cubic: Group**	**1.023**	**0.326**	**45262**	**3.134**	**0.001**
**CV: Group**	−**1.149**	**0.2**	**1086**	−**5.738**	**<0.001**
**MV: Group**	−**1.386**	**0.202**	**1086**	−**6.839**	**<0.001**
Linear: CV: Group	−0.459	0.325	45262	−1.412	0.157
**Linear: MV: Group**	−**0.701**	**0.333**	**45262**	−**2.106**	**0.035**
**Quadratic: CV: Group**	**3.59**	**0.338**	**45262**	**10.616**	**<0.001**
**Quadratic: MV: Group**	**2.626**	**0.341**	**45262**	**7.683**	**<0.001**
Cubic: CV: Group	−0.114	0.334	45262	−0.342	0.732
Cubic: MV: Group	0.481	0.34	45262	1.413	0.157

Formula: log-odds ∼ (Linear + Quadratic + Cubic) × Condition × Group + [(Linear + Quadratic + Cubic)| Subject] + (1| Item). Conditions: Unrelated verbs (UV), closely related verbs (CV), moderately related verbs (MV). TD, typical development; DS, Down syndrome; *SE*, standard error; *df*, degrees of freedom. Bold values indicate significant effects.

The middle and lower panels of Figure 2 show the probability of fixation in each condition (left) and the modeled data (right) for the TD and DS groups, respectively. Preliminary examination revealed that the TD group had more fixations in the CV and MV conditions after the verb presentation and in the UV condition after the noun presentation. The DS group presented an increase in fixation after the verb in the CV condition and after the noun in the MV and UV conditions.

The growth curve analysis revealed that in the TD group, the CV and MV had a greater and sharper increase in looking (seen in interaction with the quadratic term) than the UV condition. The positive slope of the interaction of the groups with the cubic term suggests that in the UV condition, the DS group looked more at the target and looked away faster than the TD group. The interaction of the groups with both conditions (CV and MV) indicated that in the control group, there was a greater difference between the predictive and the non-predictive conditions than in the DS group; this pattern of looking was sharper in the TD than in the DS group (seen in interaction with the quadratic term). Finally, the interaction of the groups with the linear term suggests that the difference between the MV and UV conditions increases faster over time in the control group than in the DS group.

The cluster-based permutation analysis revealed that children with TD looked more in the CV than in the UV condition in two time clusters: from 1,800 to 2,000 ms (*t*_*cluster*_ = 7.384, *t*_*max*_ = 2.747, *p* = 0.025) and from 2,600 to 5,700 ms (*t*_*cluster*_ = 168.421, *t*_*max*_ = 9.571, *p* < 0.001). They also looked more in the MV than in the UV condition in three time clusters: from 2,900 to 4,300 ms (*t*_*cluster*_ = 57.854, *t*_*max*_ = 5.806, *p* < 0.001), from 4,700 to 5,200 ms (*t*_*cluster*_ = 17.835, *t*_*max*_ = 7.735, *p* < 0.001), and from 5,400 to 5,700 ms (*t*_*cluster*_ = 14.468, *t*_*max*_ = 4.616, *p* < 0.001). The TD group looked more than chance level in the CV condition in two time clusters: from 1,800 to 2,100 ms (*t*_*cluster*_ = 12.211, *t*_*max*_ = 3.514, *p* = 0.003) and from 2,600 to 6,000 ms (*t*_*cluster*_ = 322.759, *t*_*max*_ = 14.883, *p* < 0.001). They also looked more than chance level in the MV condition in two clusters: from 2,400 to 2,500 ms (*t*_*cluster*_ = 6.236, *t*_*max*_ = 3.124, *p* = 0.039) and from 2,900 to 600 ms (*t*_*cluster*_ = 146.690, *t*_*max*_ = 7.964, *p* < 0.001). The TD group looked more than chance level in the UV condition from 4,600 to 5,400 ms (*t*_*cluster*_ = 27.553, *t*_*max*_ = 4.082, *p* < 0.001).

Participants with DS looked more in the UV than in the CV condition from 1,400 to 1,500 ms (*t*_*cluster*_ = 5.589, *t*_*max*_ = 2.113, *p* = 0.006), but more in the CV than in the UV condition from 3,800 to 3,900 ms (*t*_*cluster*_ = 6.340, *t*_*max*_ = 3.402, *p* = 0.002), and from 4,200 to 4,400 ms (*t*_*cluster*_ = 7.923, *t*_*max*_ = 2.808, *p* < 0.001). They also looked more in the UV than in the MV condition from 1,300 to 1,500 ms (*t*_*cluster*_ = 8.562, *t*_*max*_ = 3.346, *p* < 0.001). They looked more at the target than chance level in the CV condition from 2,800 to 6,000 ms (*t*_*cluster*_ = 126.342, *t*_*max*_ = 7.0733, *p* < 0.001), and more at the target than chance level in the MV condition from 5,200 to 6,000 ms (*t*_*cluster*_ = 24.491, *t*_*max*_ = 3.665, *p* < 0.001). They looked more at the target than chance level in the UV condition in three time clusters: from 1,300 to 1,600 ms (*t*_*cluster*_ = 12.014, *t*_*max*_ = 3.397, *p* < 0.001), from 3,600 to 4,000 ms (*t*_*cluster*_ = 11.827, *t*_*max*_ = 2.542, *p* < 0.001), and from 4,800 to 6,000 ms (*t*_*cluster*_ = 70.208, *t*_*max*_ = 8.033, *p* < 0.001).

### Factors influencing prediction

The binomial mixed-effect analysis showed that the reference model replicated the main results of the temporal analysis (Table 5); the CV condition, but not the MV condition, had more predictive looks than the UV condition. The fixation probability was higher in the predictive conditions (CV and MV) than in the non-predictive ones (UV).

**TABLE 5:** Model of the average prediction window for the DS group.

Fixed effects	β	*SE*	*z*	*P*
Intercept	−0.135	0.116	−1.167	0.243
**CV**	**0.458**	**0.115**	**3.986**	**<0.001**
MV	−0.119	0.161	−0.738	0.460

Formula: log odds ∼ Condition + (Cond| Subject) + (1| Item). Conditions: Unrelated verbs (UV), high-related verbs (CV), low-related verbs (MV). DS, Down syndrome; *SE*, standard error; *df*, degrees of freedom. Bold values indicate significant effects.

The model comparison found that including the factors of chronological age or production did not improve the fit (Table 6). Furthermore, all Bayes factors were <0.001, suggesting that the null hypothesis should be accepted. Thus, neither chronological age nor production were related to the prediction effect.

**TABLE 6:** Fit comparison of demographic models for the DS group.

Fixed effect structure	Ln(L)	*X* ^2^	*p*
Condition	−28103		
Condition × Chronological age	−28100	6.745	0.080
**Condition** × **Mental age**	−**28097**	**12.717**	**0.005**
Condition × Production	−28102	2.098	0.552
**Condition** × **Association strength**	−**28065**	**75.608**	**<0.001**

All models were compared directly with the reference model (*df* = 3). The dependent variable was the log odds ratio of fixation. The random structures were the subject and the slope of the condition, and the intercept of the Item. Condition: unrelated verb, closely related verb, moderately related verb. DS, Down syndrome. Ln(L), −2 times log-likelihood. Bold values indicate significant effects.

In contrast, mental age significantly improved the fit of the model (Table 6). Further exploration of the mental age model showed a significant interaction between the MV condition and mental age, indicating that the differences between the UV and MV conditions increase with mental age.

Notably, the slope of the results was negative (Table 7), indicating that participants looked less in the MV than in the UV condition as mental age increased. This result should be taken with caution because the Bayes factor provides evidence in favor of the null hypothesis (BF < 0.001).

**TABLE 7:** Model for mental age exploration in the DS group.

Fixed effects	β	*SE*	*z*	*p*
Intercept	−0.042	0.158	−0.266	0.789
**CV**	**0.263**	**0.168**	**1.568**	**0.116**
**MV**	−**0.656**	**0.198**	−**3.315**	**<0.001**
Mental age	0.333	0.393	0.848	0.396
CV: Mental age	−0.697	0.456	−1.525	0.127
**MV: Mental age**	−**1.916**	**0.538**	−**3.563**	**<0.001**

Formula: log odds ∼ Condition × Mental age + (Cond| Subject) + (1| Item). *SE*, standard error; *df*, degrees of freedom. Bold values indicate significant effects.

The association strength between the verb and the expected noun also improved the fit of the model (Table 6), and it provided strong evidence in favor of the alternative hypothesis (BF = 2.98e + 10). Exploration of the model including association strength showed an interaction between association strength and both the CV and MV conditions, with a positive slope (Table 8). This result indicates that both predictive conditions showed more predictive looks than the UV condition with higher association strength.

**TABLE 8:** Model for association strength exploration in the DS group.

Fixed effects	β	*SE*	*z*	*p*
Intercept	−0.153	0.114	−1.341	0.179
CV	0.191	0.132	1.446	0.148
**MV**	**0.707**	**0.199**	**3.553**	**<0.001**
**Association Strength**	−**0.434**	**0.197**	−**2.202**	**0.027**
**CV: Association Strength**	**1.019**	**0.216**	**4.716**	**<0.001**
**MV: Association Strength**	**2.424**	**0.34**	**7.115**	**<0.001**

Formula: log odds ∼ Condition × Association Strength + (Cond| Subject) 1| Item). Conditions: High-related verbs (CV), low-related verbs (MV). *SE*, standard error; *df*, degrees of freedom. Bold values indicate significant effects.

## Discussion

We tested the prediction ability of young people with DS and a control group of children with TD, paired by verbal mental age of around 5 years, based on the relationship between a heard verb and a depicted pair of images representing target and distractor nouns. We also tested a group of adults with TD to corroborate the prediction effect expected in the other two groups. We presented three types of relationships between verbs and nouns embedded in sentences: closely related verb (CV; e.g., *to read—book*), moderately related verb (MV; e.g., *to wait—bus*), and unrelated verb (UV; e.g., *to arrive—dog*). We hypothesized that adults and children with TD would predict the intended target in both closely and moderately related sentences but not in unrelated pairs. In the case of participants with DS, we expected to capture prediction only with closely related verbs but not with moderately related or unrelated verbs. Finally, we expected that vocabulary production would play a significant role in prediction by participants with DS.

Our results corroborate our hypothesis for adults and children with TD. Both groups could anticipate the target before it was named, based on the level of relationship between the verb and the noun. In the case of young people with DS, we found an ability to predict only in closely related sentences, confirming their need for a high degree of relationship between verbs and nouns. We also found that their ability to predict was slow compared to children with TD: they took about 200 ms longer to anticipate the target noun. In all cases, preference for the labeled noun at the end of the noun window confirmed that participants followed the task. Our last hypothesis, positing a relationship between the level of the productive vocabulary of people with DS and their predictive ability, was not confirmed.

Our results show that participants with DS could anticipate the subsequent noun only in sentence constructions with a closely related verb, not in those with a moderately related verb, while the TD group showed linguistic anticipation skills with both closely and moderately related verbs. These results support the idea that different factors are involved in prediction, depending on the degree of relationship between the context and the upcoming word, as proposed by the theoretical prediction models (Pickering and Gambi, 2018). A higher degree of association between the verb and the noun makes the generation of linguistic predictions more likely, even though both sentence constructions are possible at the grammatical level and also predictable. The development of these differential factors associated with moderately related verbs could be delayed or impaired in DS participants but not in those with TD.

In their prediction theory, Pickering and Gambi (2018) postulate two prediction mechanisms: prediction-by-association and prediction-by-production. Although both predictive mechanisms are involved in the experimental condition, the sentences were designed to require different uses of each mechanism. The prediction-by-association mechanism is based on spreading activation between related concepts, is automatic, and uses fewer cognitive resources. Sentence prediction with a close verb-noun relationship is assumed to be supported mainly by this mechanism because the activation spreads strongly from the verb to the noun. The activation of the target may also produce lateral inhibition in unrelated elements of the lexicon (Chow et al., 2016; Angulo-Chavira and Arias-Trejo, 2021): in this case, the distractor. Our results in the closely related condition show that these mechanisms are relatively preserved in participants with DS, which is consistent with previous studies showing spreading activation between related nouns in this population (Barrón-Martínez and Arias-Trejo, 2020; Barrón-Martínez et al., 2020). Nevertheless, people with DS seem to present weak connections between related concepts: the magnitude and velocity of the predictions are less in the DS group than in the TD group. This explanation is plausible, at least for the connection between verbs and nouns, because children with DS present a similar spreading activation between nouns as their mental age peers (Barrón-Martínez and Arias-Trejo, 2020; Barrón-Martínez et al., 2020).

By contrast, prediction-by-production is efficient because it uses linguistic and non-linguistic contextual information and interaction with the speaker to make inferences about their intentions. This system is slow, uses a high level of cognitive resources, and is optional (Pickering and Gambi, 2018). We assume that our moderately related condition depends on prediction-by-production because participants needed to rely more on visual information to predict the target.^4^ For example, there is more variability in the possible direct objects connected to the verb *fix* than to the verb *sweep*, which is closely related to *broom*; participants are thus forced to look for a *fixable* object and discard all *unfixable* objects based on the picture displayed (e.g., *washing machine* vs. *watermelon*). Adults and children with TD predicted the moderately related verb condition; however, in the TD group, they did so less in this condition than in the closely related condition, suggesting that the moderately related condition is harder to process, in line with the prediction-by-production hypothesis. Note that adults did not present a clear difference between the two, indicating that prediction-by-production improves during development, at least for a syntactically simple sentence with common words. It is possible that the prediction in the closely related and moderately related conditions behaved asymptotically, as in associative learning models (Plaut and Booth, 2000; Kapatsinski, 2021). This asymptotic behavior contributes to maintaining a degree of uncertainty (Kapatsinski, 2021) and when the associations are weak (Plaut and Booth, 2000), there is a delay in approaching the asymptotic point, as seen in the TD children and adults in our study. Participants with DS possibly did not have enough resources to predict the moderately related condition. Since prediction-by-production is optional (Huettig, 2015; Huettig and Mani, 2016; Pickering and Gambi, 2018), the language system prioritizes the comprehension of bottom-up information over top-down prediction.

The question then arises as to what resources are necessary for people with DS to use prediction-by-production. To answer this question, we explored variables that could explain the individual difference in prediction, particularly chronological age, mental age, association strength, and production. We measured the influence of chronological age in prediction skills because older participants have more experience with language than younger ones; however, it seems that the ability to predict a referent in highly or less highly semantically related environments does not underlie this factor. This result does not mean that prediction is not dependent on experience in people with DS; in fact, the prediction of highly semantically related information indicates that they need very common word pairs to make predictions. The sentences with close relationships were also those that our plausibility study found to have higher probabilities of being heard. The association strength between the verb and the noun also facilitates prediction regardless of the condition. Thus, the frequency of the sentences and the frequency of the relationships may contribute to the prediction of a noun. Less common combinations of verbs and nouns also diminish predictive ability in young people with DS.

We also found that mental age influences prediction in people with DS in an unexpected direction: participants with DS with greater mental age looked less at the target in the MV condition. This result is contrary to that of Arias-Trejo et al. (2019), who found a positive correlation between mental age and the predictive ability of people with DS. Differences in the mental age evaluation might explain this discrepancy. Arias-Trejo et al. (2019) computed mental age by evaluating verbal and non-verbal cognitive domains. In the present study, mental age was based on comprehension ability. In other words, if comprehension skills do not determine the ability of people with DS to predict the upcoming noun, then more general cognitive skills may do.

Associative models show that an increase in vocabulary produces difficulties in word recognition because of the competition and addition of weak associations to the lexicon (Ramscar et al., 2014). This difficulty might be present in the predictive recovery of words. It is possible that people with DS had such difficulties related to the addition of new words to the lexicon. One mechanism that helps overcome competition problems is inhibition (e.g., McClelland and Elman, 1986), a mechanism developed in early childhood (Chow et al., 2016, 2019). People with DS may suppress weak associations to avoid the interference produced by the competition that increases with cognitive development. This interpretation is supported by the observation that people with DS predict better when the association strength is higher. Nevertheless, this is a speculative interpretation and should be taken with caution, not only because our experiment was not designed to prove this point but also because the Bayes factor provides evidence against the influence of verbal mental age on prediction skills.

It is hypothesized that predictions are made by the production system (Dell and Chang, 2014; Huettig, 2015; Huettig and Janse, 2016; Pickering and Gambi, 2018). For example, participants who scored better on productive vocabulary tests were those who also presented better linguistic anticipation skills (Mani and Huettig, 2012; Mani et al., 2016). In the present study, we found no influence of production, either in the closely related or moderately related verb conditions, in any group of participants.

The lack of a relationship between production and prediction could be interpreted as the production system not being involved in the generation of top-down predictions; however, this is unlikely in light of previous evidence (Martin et al., 2013; Dell and Chang, 2014; Huettig, 2015; Huettig and Janse, 2016; Pickering and Gambi, 2018). A second explanation is in the use of resources in prediction-by-production: people with DS have several cumulative factors that can hinder top-down predictions. Working memory problems and processing speed in people with DS are likely to interfere with the ability to predict upcoming linguistic information (Huettig and Janse, 2016; Ito et al., 2018). For example, Huettig and Janse (2016) found more predictive eye movements in the visual world paradigm in people with better working memory and faster processing speed. Participants with DS tended to have poor reading skills, which could hinder their ability to predict, as reported by Mishra (2012) for adults with low literacy and (Huettig and Brouwer, 2015) for Dutch adults with dyslexia. Thus, the lack of a relationship between production and top-down predictions in people with DS may be better explained by limitations in general cognition.

### Limitations and future studies

The present study describes some prediction processes in people with DS; however, it is important to consider some of the study’s limitations. First, the sample of participants is small; it is difficult to generalize our results to all populations with DS since there is a high degree of variability in their cognitive profiles. The sample size also affects the fixation data. There are unexpected but significant differences across the trial: a slight preference for the unrelated condition in the pre-verb and verb windows in the DS group and a slight preference in the pre-verb window for the closely related verb condition. We assume that these differences result from the small sample because there is no consistency in the presentation of these clusters across groups or conditions; it can thus be interpreted as a random preference created by the high variability of our data.

Another limitation is that we evaluated only receptive and expressive vocabulary because the COVID-19 pandemic required us to administer the assessments online. Future studies must explore more general cognitive skills, such as working memory and processing speed, to better explain the factors underlying DS prediction. Receptive vocabulary as a measure of verbal mental age could also be insufficient; further research should measure additional language skills or general cognitive development.

People with DS also have a high prevalence of nystagmus, which affects ocular control (Mathan et al., 2022). Given the online nature of our study, we relied on parents for information about possible problems with vision and hearing. Although this bias would be a constant in the within-subject comparisons, it is necessary to consider the problem in the between-subject comparisons and also consider more robust measures of ocular problems in the population with DS.

## Conclusion

This study evaluated prediction skills in people with DS using a preferential-looking task. It provides evidence that young people with DS can anticipate upcoming information based on the semantic relatedness between a verb and a noun. Participants with DS predicted nouns in closely related verb-noun pairs but not in pairs that were only moderately related and in which they needed visual context to generate the prediction. These effects are not explained by chronological age, mental age, or productive vocabulary. These results suggest that in people with DS, prediction is driven by association; this offers clues about how people in this group process and extract information from speech and in context. By studying the mechanism that allows this, we can better understand how this population uses it to learn more rapidly in situations varying in context and how established predictions can be used to promote learning. Our findings support an ecological and feasible evaluation tool for the systematic measurement of lexical prediction in people with DS, useful for understanding the cognitive mechanisms of lexical prediction and how these mechanisms can be strengthened through the implementation of stimulation programs.

## Data availability statement

The raw data supporting the conclusions of this article will be made available by the authors, without undue reservation.

## Ethics statement

The studies involving human participants were reviewed and approved by the Comité de Ética, Facultad de Psicología, UNAM. Written informed consent to participate in this study was provided by the participants’ legal guardian/next of kin.

## Author contributions

AA-C: conception of the original idea, experimental design, data analysis, data processing, stimulus creation, revision, and critical writing of the manuscript. AC-F: experimental design, conceptualization of the final experimental design, creation of stimuli, data processing, search, and attention of participants, and writing of the manuscript. JB-M: search and attention of participants, and writing of the manuscript. NA-T: funding acquisition, administration and supervision of the project, conceptualization of experimental design, revision, and critical writing of the manuscript. All authors contributed to the article and approved the submitted version.

## Footnotes

1 Studies have found, however, that in the early stages of development, production is equivalent in groups matched by mental age (Galeote et al., 2011; Checa et al., 2016).

2 Throughout this text we will use the words *association* and *relationship* as synonyms.

3 The auditory sentences were manipulated by adding pauses so that the verbs and expected nouns in all of the sentences were heard at the same positions on the timeline, as in previous studies with children (Mani and Huettig, 2012, 2014; Arias-Trejo et al., 2019). This presentation is advantageous for subjects with processing speed problems, and it also provides a specific period for prediction. We thus expected the predictive look in the verb window for the CV and MV conditions, and the look to the named noun in the noun window for all conditions.

4 Prediction-by-association could be involved, since there is a degree of association; however, its involvement should be small, since our free association task asked for a verb. It is possible that in typical association norms participants do not even mention these verbs.

## References

[B1] Altmann G. T. M. Kamide Y. (1999). Incremental interpretation at verbs: Restricting the domain of subsequent reference. *Cognition* 73 247–264. 10.1016/S0010-0277(99)00059-1 10585516

[B2] Alva-Canto E. A. (2001). Cómo usan los niños las palabras. El uso de los derivados de las palabras en el lenguaje espontáneo de los niños en interacción libre entre iguales. *Facultad de Psicología, Univ. Nacional Autónoma de México* 1, 19–173.

[B3] Anderson J. R. (2013). A spreading activation theory of memory. *Read. Cogn. Sci. Perspect. Psychol. Artifi. Intellig.* 2013 137–154. 10.1016/B978-1-4832-1446-7.50016-9

[B4] Andreou G. Katsarou D. (2016). *Semantic Processing In Children With Down Syndrome.* 59–66. Available online at: 10.26262/istal.v21i0.5208 (accessed January 23, 2022).

[B5] Angulo-Chavira A. Q. Arias-Trejo N. (2021). Mediated semantic priming interference in toddlers as seen through pupil dynamics. *J. Exp. Child Psychol.* 208:105146. 10.1016/j.jecp.2021.105146 33862526

[B6] Arias-Trejo N. Angulo-Chavira A. Q. Barrón-Martínez J. B. (2019). Verb-mediated anticipatory eye movements in people with down syndrome. *Int. J. Lang. Commun. Dis.* 54 756–766. 10.1111/1460-6984.12473 30983122

[B7] Arias-Trejo N. Falcón A. Alva-Canto E. A. (2013). The gender puzzle: Toddlers’ use of articles to access noun information. *Psicologica* 34 1–23.

[B8] Barr D. J. (2008). Analyzing ‘visual world’ eyetracking data using multilevel logistic regression. *J. Memory Lang.* 59 457–474. 10.1016/j.jml.2007.09.002

[B9] Barrón-Martínez J. B. Arias-Trejo N. (2020). Perceptual similarity effect in people with down syndrome. *Int. J. Dev. Disabili.* 68 182–189. 10.1080/20473869.2020.1729016 35309697 PMC8928852

[B10] Barrón-Martínez J. B. Arias-Trejo N. (2022). Perceptual similarity effect in people with down syndrome. *Int. J. Dev. Disabili.* 68 182–189. 10.1080/20473869.2020.1729016 PMC8928852 35309697

[B11] Barrón-Martínez J. B. Arias-Trejo N. Salvador-Cruz J. (2020). Associative lexical relationships in children with down syndrome. *Int. J. Disabili. Dev. Educ.* 2020 1–13. 10.1016/j.jcomdis.2020.105975 32088412

[B12] Borovsky A. Elman J. L. Fernald A. (2012). Knowing a lot for one’s age: Vocabulary skill and not age is associated with anticipatory incremental sentence interpretation in children and adults. *J. Exp. Child Psychol.* 112 417–436. 10.1016/j.jecp.2012.01.005 22632758 PMC3374638

[B13] Campanelli L. van Dyke J. A. Marton K. (2018). *CUNY Academic Works The Modulatory Effect of Expectations on Memory Retrieval During Sentence Comprehension How Does Access To This Work Benefit You? Let Us Know! The Modulatory Effect of Expectations on Memory Retrieval During Sentence Comprehension.* New York City, NY: City University New York Graduated Center.

[B14] Chapman R. S. (2006). Language learning in down syndrome: The speech and language profile compared to adolescents with cognitive impairment of unknown origin. *Down Syndrome Res. Pract. J. Sarah Duffen Centre Univ. Portsmouth* 10 61–66. 10.3104/reports.306 16869363

[B15] Chapman R. S. Seung H. K. Schwartz S. E. Bird E. K. (2000). Predicting language production in children and adolescents with down syndrome: The role of comprehension. *J. Speech Lang. Hear. Res.* 43 343–350. 10.1044/jslhr.4302.340 10757688

[B16] Checa E. Galeote M. Soto P. (2016). The Composition of early vocabulary in Spanish children with down syndrome and their peers with typical development. *Am. J. Speech Lang. Pathol.* 25 605–619. 10.1044/2016_AJSLP-15-0095 27893086

[B17] Cheung R. W. Hartley C. Monaghan P. (2022). Receptive and expressive language ability differentially support symbolic understanding over time: Picture comprehension in late talking and typically developing children. *J. Exp. Child Psychol.* 214:105305. 10.1016/j.jecp.2021.105305 34653634

[B18] Chow J. Aimola Davies A. M. Fuentes L. J. Plunkett K. (2016). Backward semantic inhibition in toddlers. *Psychol. Sci.* 27 1312–1320. 10.1177/0956797616659766 27519530

[B19] Chow J. Aimola Davies A. M. Fuentes L. J. Plunkett K. (2019). The vocabulary spurt predicts the emergence of backward semantic inhibition in 18-month-old toddlers. *Dev. Sci.* 22:12754. 10.1111/desc.12754 30248216

[B20] Cognition Run (2021). *Cogntion Run Cognitive Experiments Online.* Available online at: https://www.cognition.run/ (accessed October 10, 2021).

[B21] Collins A. M. Loftus E. F. (1975). A spreading-activation theory of semantic processing. *Psychol. Rev.* 82 407–428. 10.1037/0033-295X.82.6.407

[B22] Dell G. S. (1986). A spreading-activation theory of retrieval in sentence production. *Psychol. Rev.* 93 283–321. 10.1037/0033-295X.93.3.283 3749399

[B23] Dell G. S. Chang F. (2014). The p-chain: Relating sentence production and its disorders to comprehension and acquisition. *Philos. Trans. R. Soc. Biol. Sci.* 369:394. 10.1098/rstb.2012.0394 24324238 PMC3866424

[B24] DeLong C. Nessler C. Wright S. Wambaugh J. (2015). Semantic feature analysis: Further examination of outcomes. *Am. J. Speech Lang. Pathol.* 24 1–14. 10.1044/2015_AJSLP-14-0155 26384102

[B25] Dierssen M. (2012). Down syndrome: The brain in trisomic mode. *Nat. Rev. Neurosci.* 13 844–858. 10.1038/nrn3314 23165261

[B26] Dodd B. Thompson L. (2001). Speech disorder in children with Down’s syndrome. *J. Intellect. Disabili. Res.* 45 308–316. 10.1046/j.1365-2788.2001.00327.x 11489052

[B27] Eadie P. A. Fey M. E. Douglas J. M. Parsons C. L. (2002). Profiles of grammatical morphology and sentence imitation in children with specific language impairment and down syndrome. *J. Speech Lang. Hear. Res.* 45 720–732. 10.1044/1092-4388(2002/058) 12199402

[B28] Federmeier K. D. (2007). Thinking ahead: The role and roots of prediction in language comprehension. *Psychophysiology* 44 491–505. 10.1111/j.1469-8986.2007.00531.x 17521377 PMC2712632

[B29] Federmeier K. D. Kutas M. (1999). A rose by any other name: long-term memory structure and sentence processing. *J. Memory Lang.* 41 469–495. 10.1006/jmla.1999.2660

[B30] Ferretti T. R. McRae K. Hatherell A. (2001). Integrating verbs, situation schemas, and thematic role concepts. *J. Memory Lang.* 44 516–547. 10.1006/jmla.2000.2728

[B31] Fine A. B. Jaeger T. F. Farmer T. A. Qian T. (2013). Rapid expectation adaptation during syntactic comprehension. *PLoS One* 8:77661. 10.1371/journal.pone.0077661 24204909 PMC3813674

[B32] Galeote M. Sebastián E. Checa E. Rey R. Soto P. (2011). The development of vocabulary in Spanish children with down syndrome: Comprehension, production, and gestures. *J. Intellect. Dev. Disabili.* 36 184–196. 10.3109/13668250.2011.599317 21843033

[B33] Gambi C. Gorrie F. Pickering M. J. Rabagliati H. (2018). The development of linguistic prediction: Predictions of sound and meaning in 2- to 5-year-olds. *J. Exp. Child Psychol.* 173 351–370. 10.1016/j.jecp.2018.04.012 29793772

[B34] Hsu C. F. (2019). Contextual effects on semantic grouping in individuals with down syndrome. *Int. J. Dev. Disabilit.* 65 65–72. 10.1080/20473869.2017.1353659 34141325 PMC8115440

[B35] Huettig F. (2015). Four central questions about prediction in language processing. *Brain Res.* 1626 118–135. 10.1016/j.brainres.2015.02.014 25708148

[B36] Huettig F. Brouwer S. (2015). Delayed anticipatory spoken language processing in adults with dyslexia - evidence from eye-tracking. *Dyslexia* 21 97–122. 10.1002/dys.1497 25820191

[B37] Huettig F. Janse E. (2016). Individual differences in working memory and processing speed predict anticipatory spoken language processing in the visual world. *Lang. Cogn. Neurosci.* 31 80–93. 10.1080/23273798.2015.1047459

[B38] Huettig F. Mani N. (2016). Is prediction necessary to understand language? Probably not. *Lang. Cogn. Neurosci.* 31 19–31. 10.1080/23273798.2015.1072223

[B39] Ito A. Corley M. Pickering M. J. (2018). A cognitive load delays predictive eye movements similarly during L1 and L2 comprehension. *Bilingualism* 21 251–264. 10.1017/S1366728917000050

[B40] Ito K. Speer S. R. (2008). Anticipatory effects of intonation: Eye movements during instructed visual search. *J. Memory Lang.* 58 541–573. 10.1016/j.jml.2007.06.013 19190719 PMC2361389

[B41] Iverson J. M. Longobardi E. Caselli M. C. (2003). Relationship between gestures and words in children with Down’s syndrome and typically developing children in the early stages of communicative development. *Int. J. Lang. Commun. Dis.* 38 179–197. 10.1080/1368282031000062891 12745936

[B42] Kamide Y. Altmann G. T. M. Haywood S. L. (2003). The time-course of prediction in incremental sentence processing: Evidence from anticipatory eye movements. *J. Memory Lang.* 49 133–156. 10.3389/fpsyg.2016.00150 27014107 PMC4786575

[B43] Kapatsinski V. (2021). Learning fast while avoiding spurious excitement and overcoming cue competition requires setting unachievable goals: Reasons for using the logistic activation function in learning to predict categorical outcomes. *Lang. Cogn. Neurosci.* [Preprint]. 10.1080/23273798.2021.1927120

[B44] Kumin L. Councill C. Goodman M. (1994). A longitudinal study of the emergence of phonemes in children with down syndrome. *J. Commun. Disord.* 27 293–303. 10.1016/0021-9924(94)90019-1 7876409

[B45] Kuperberg G. R. Jaeger T. F. (2016). What do we mean by prediction in language comprehension? *Lang. Cogn. Neurosci.* 31 32–59. 10.1080/23273798.2015.1102299 27135040 PMC4850025

[B46] Laws G. Briscoe J. Ang S. Y. Brown H. Hermena E. Kapikian A. (2015). Receptive vocabulary and semantic knowledge in children with SLI and children with down syndrome. *Child Neuropsychol.* 21 490–508. 10.1080/09297049.2014.917619 24830646

[B47] Lubec G. Engidawork E. (2002). The brain in down syndrome (trisomy 21). *J. Neurol.* 249 1347–1356. 10.1007/s00415-002-0799-9 12382149

[B48] Lukyanenko C. Fisher C. (2016). Where are the cookies? Two- and three-year-olds use number-marked verbs to anticipate upcoming nouns. *Cognition* 146 349–370. 10.1016/j.cognition.2015.10.012 26513355 PMC4673033

[B49] Makowski D. Ben-Shachar M. Lüdecke D. (2019). bayestestR: Describing effects and their uncertainty, existence and significance within the bayesian framework. *J. Open Source Softw.* 4:1541. 10.21105/joss.01541

[B50] Mani N. Huettig F. (2012). Prediction during language processing is a piece of cake-but only for skilled producers. *J. Exp. Psychol. Hum. Percept. Perform.* 38 843–847. 10.1037/a0029284 22774799

[B51] Mani N. Huettig F. (2014). Word reading skill predicts anticipation of upcoming spoken language input: A study of children developing proficiency in reading. *J. Exp. Child Psychol.* 126 264–279. 10.1016/j.jecp.2014.05.004 24955519

[B52] Mani N. Daum M. M. Huettig F. (2016). ‘Proactive’ in many ways: Developmental evidence for a dynamic pluralistic approach to prediction. *Quart. J. Exp. Psychol.* 69 2189–2201. 10.1080/17470218.2015.1111395 26595092

[B53] Maris E. Oostenveld R. (2007). Nonparametric statistical testing of EEG- and MEG-data. *J. Neurosci. Methods* 164 177–190. 10.1016/j.jneumeth.2007.03.024 17517438

[B54] Martin C. D. Branzi F. M. Bar M. (2018). Prediction is production: The missing link between language production and comprehension. *Sci. Rep.* 8 1–9. 10.1038/s41598-018-19499-4 29348611 PMC5773579

[B55] Martin G. E. Losh M. Estigarribia B. Sideris J. Roberts J. (2013). Longitudinal profiles of expressive vocabulary, syntax and pragmatic language in boys with fragile X syndrome or down syndrome. *Int. J. Lang. Commun. Dis.* 48 432–443. 10.1111/1460-6984.12019 23889838 PMC3926422

[B56] Martin K. I. Ellis N. C. (2012). The roles of phonological short-term memory and working memory in L2 grammar and vocabulary learning. *Stud. Second Lang. Acquisit.* 34 379–413. 10.1017/S0272263112000125

[B57] Martin N. A. (2010b). *ROWPVT-4: Receptive One-Word Picture Vocabulary Test.* Novato, CA: ATP: Assesment a division of Academic Therapy of Publications.

[B58] Martin N. A. (2010a). *EOWPVT-4: Expressive One-Word Picture Vocabulary Test.* Novato, CA: ATP: Assesment a division of Academic Therapy of Publications.

[B59] Mathan J. J. Simkin S. K. Gokul A. McGhee C. N. J. (2022). Down syndrome and the eye: Ocular characteristics and ocular assessment. *Survey Ophthalmol.* [Preprint]. 10.1016/j.survophthal.2022.03.006 35367480

[B60] McClelland J. L. Elman J. L. (1986). The TRACE model of speech perception. *Cogn. Psychol.* 18 1–86. 10.1016/0010-0285(86)90015-0 3753912

[B61] McRae K. Ferretti T. R. Amyote L. (1997). Thematic roles as verb-specific concepts. *Lang. Cogn. Proc.* 12 137–176. 10.1080/016909697386835

[B62] Michael S. E. Ratner N. B. Newman R. (2012). Verb comprehension and use in children and adults with down syndrome. *J. Speech Lang. Hear. Res.* 55 1736–1749. 10.1044/1092-4388(2012/11-0050) 22992705

[B63] Mirman D. (2014). *Growth Curve Analysis and Visualization Using R.* Taylor & Francis: Chapman & Hall.

[B64] Mishra R. K. (2012). Spoken language-mediated anticipatory eyemovements are modulated by reading ability - evidence from Indian low and high literates. *J. Eye Move. Res.* 5 1–10. 10.16910/jemr.5.1.3

[B65] Næss K. A. B. (2022). A randomized trial of the digital down syndrome languageplus (DSL+) vocabulary intervention program. *Remed. Special Educ.* [Preprint]. 10.1177/07419325211058400

[B66] Næss K. A. B. Lyster S. A. Hulme C. Melby-Lervåg M. (2011). Language and verbal short-term memory skills in children with down syndrome: A meta-analytic review. *Res. Dev. Disabili.* 32 2225–2234. 10.1016/j.ridd.2011.05.014 21628091

[B67] Pelucchi B. Hay J. F. Saffran J. R. (2009). Learning in reverse: Eight-month-old infants track backward transitional probabilities. *Cognition* 113 244–247. 10.1016/j.cognition.2009.07.011 19717144 PMC2763958

[B68] Pickering M. J. Gambi C. (2018). Predicting while comprehending language: A theory and review. *Psychol. Bull.* 144 1002–1044. 10.1037/bul0000158 29952584

[B69] Plaut D. C. Booth J. R. (2000). Individual and developmental differences in semantic priming: Empirical and computational support for a single-mechanism account of lexical processing. *Psychol. Rev.* 107 786–823. 10.1037/0033-295x.107.4.786 11089407

[B70] R Core Team (2019). *R: A Language And Environment For Statistical Computing, Reference Index Version 3.5.3.*

[B71] Ramscar M. Hendrix P. Shaoul C. Milin P. Baayen H. (2014). The myth of cognitive decline: Non-linear dynamics of lifelong learning. *Top. Cogn. Sci.* 6 5–42. 10.1111/tops.12078 24421073

[B72] RealEye (2020). *RealEye Eye-Tracking System Technology Whitepaper.* Available online at: https://d48dl6cezsco5.cloudfront.net/static/doc/RealEye+-+Technical+Whitepaper+v1.1.0.pdf (accessed July 27, 2022).

[B73] Reuter T. Borovsky A. Lew-Williams C. (2019). Predict and redirect: Prediction errors support children’s word learning. *Dev. Psychol.* 55 1656–1665. 10.1037/dev0000754 31094555 PMC6876992

[B74] Ricketts J. Nation K. Bishop D. V. M. (2007). Vocabulary is important for some, but not all reading skills. *Sci. Stud. Read.* 11 235–257. 10.1080/10888430701344306

[B75] Roberts J. Long S. H. Malkin C. Barnes E. Skinner M. Hennon E. A. (2005). A comparison of phonological skills of boys with fragile X syndrome and down syndrome. *J. Speech Lang. Hear. Res.* 48 980–995. 10.1044/1092-4388(2005/067) 16411789

[B76] Roberts J. Martin G. E. Moskowitz L. Harris A. A. Foreman J. Nelson L. (2007). Discourse skills of boys with fragile X syndrome in comparison to boys with down syndrome. *J. Speech Lang. Hear. Res.* 50 475–492. 10.1044/1092-4388(2007/033) 17463242

[B77] Rommers J. Meyer A. S. Huettig F. (2015). Verbal and nonverbal predictors of language-mediated anticipatory eye movements. *Attent. Percept. Psychophys.* 77 720–730. 10.3758/s13414-015-0873-x 25795276

[B78] Rosin M. M. (1988). Communication profiles of adolescents with down syndrome. *Commun. Dis. Quart.* 12 49–64. 10.1177/152574018801200105

[B79] Schoknecht P. (2022). The interaction of predictive processing and similarity-based retrieval interference: An ERP study. *Lang. Cogn. Neurosci.* 2022 1–19. 10.1080/23273798.2022.2026421

[B80] Stefaniak N. Baltazart V. Declercq C. (2021). Processing verb meanings and the declarative/procedural model: A developmental study. *Front. Psychol.* 12:714523. 10.3389/fpsyg.2021.714523 34659028 PMC8514706

[B81] Stolt S. Lind A. Matomäki J. Haataja L. Lapinleimu H. Lehtonen L. (2016). Do the early development of gestures and receptive and expressive language predict language skills at 5;0 in prematurely born very-low-birth-weight children? *J. Commun. Dis.* 61 16–28. 10.1016/j.jcomdis.2016.03.002 26999726

[B82] van Gameren-Oosterom H. B. M. Fekkes M. Buitendijk S. E. Mohangoo A. D. Bruil J. Van Wouwe J. P. (2011). Development, problem behavior, and quality of life in a population based sample of eight-year-old children with down syndrome. *PLoS One* 6:16–20. 10.1371/journal.pone.0021879 21814560 PMC3140989

[B83] Wass S. V. Forssman L. Leppänen J. (2014). Robustness and precision: How data quality may influence key dependent variables in infant eye-tracker analyses. *Infancy* 19 427–460. 10.1111/infa.12055

[B84] Wetzels R. Matzke D. Lee M. D. Rouder J. N. Iverson G. J. Wagenmakers E. J. (2011). Statistical evidence in experimental psychology: An empirical comparison using 855 t tests. *Perspect. Psychol. Sci.* 6 291–298. 10.1177/1745691611406923 26168519

[B85] Witecy B. Penke M. (2017). Language comprehension in children, adolescents, and adults with down syndrome. *Res. Dev. Disabili.* 62 184–196. 10.1016/j.ridd.2017.01.014 28187370

[B86] Yoder P. J. Camarata S. Camarata M. Williams S. M. (2006). Association between differentiated processing of syllables and comprehension of grammatical morphology in children with down syndrome. *Am. Assoc. Mental Retardat.* 111 138–152. 10.1352/0895-8017(2006)111[138:ABDPOS]2.0.CO;2 16466285

