# Supplementary material for: Word prediction using closely and moderately related verbs in Down syndrome
Source: Front Psychol. 2022 Oct 3;13:934826. doi: 10.3389/fpsyg.2022.934826 (PMC9574260; doi:10.3389/fpsyg.2022.934826)
Supplement: Supplementary file 1 [file Table_1.DOCX]

Supplementary Material

Table S1. Target and distractor picture for each experimental sentence.

| **Sentence ID** | **Image** |
| --- | --- |
| My father swam in the… | 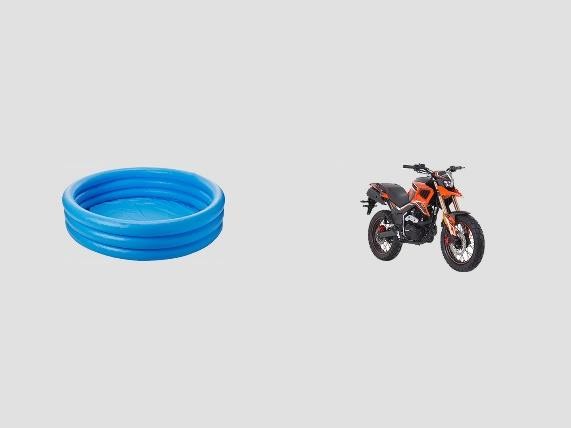 |
| In the morning, a . . . barked | 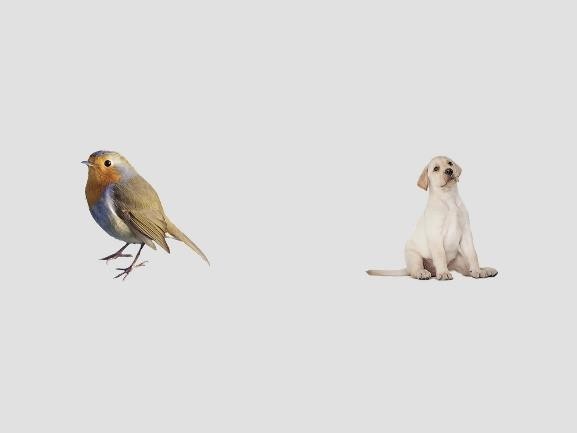 |
| The woman read the… | 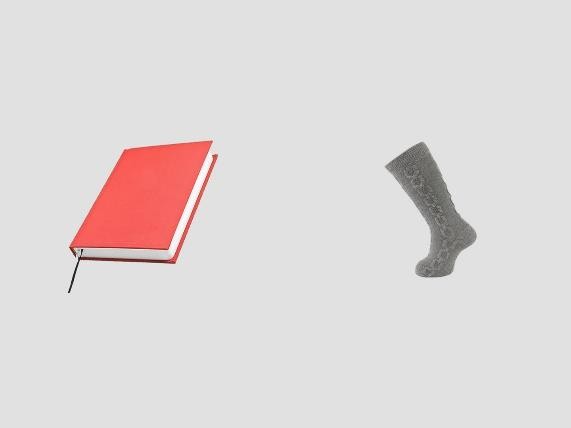 |

| My sister swept with a… | 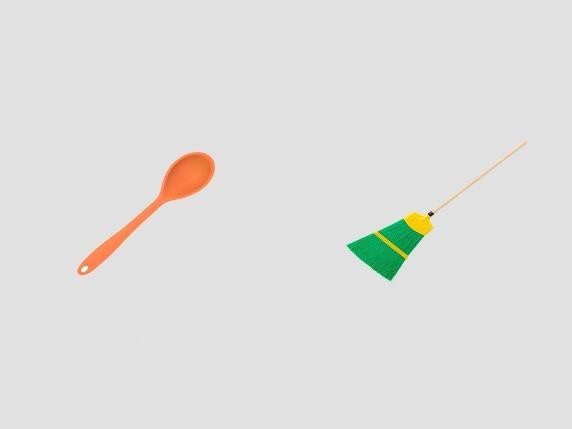 |
| --- | --- |
| My brother wrote with the… | 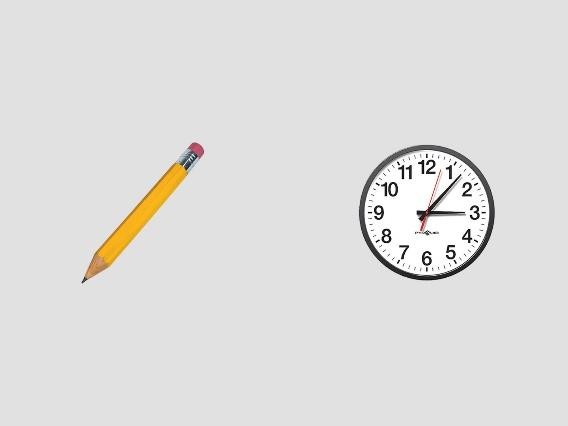 |
| My cousin cut himself with a… | 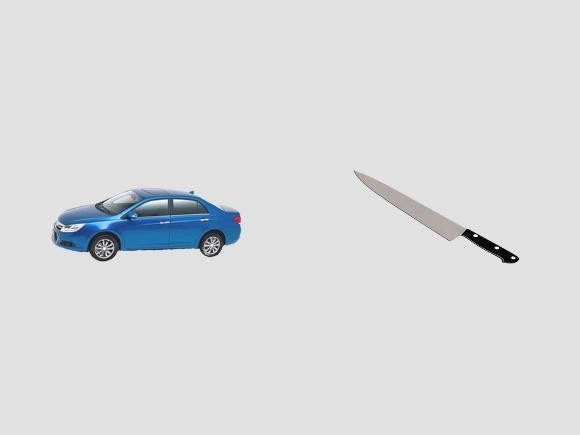 |
| My aunt ate a… | 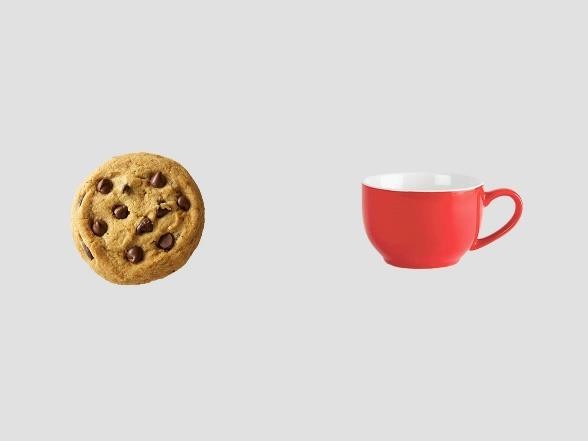 |

| My mother talked on the… | 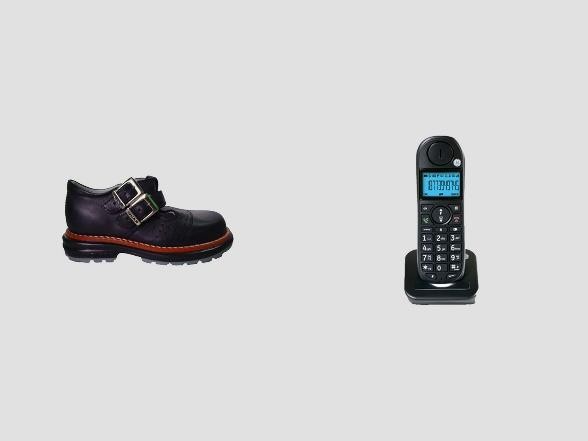 |
| --- | --- |
| My friend fell asleep in the… | 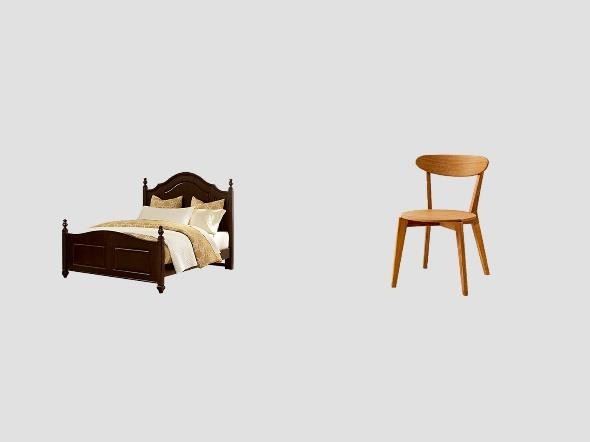 |
| My partner drank the… | 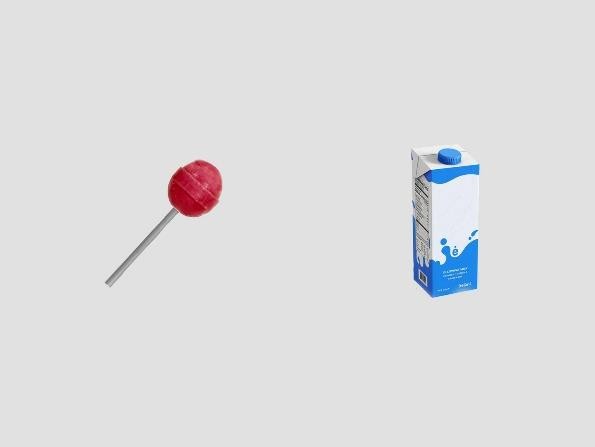 |
| My uncle bit a(n)… | 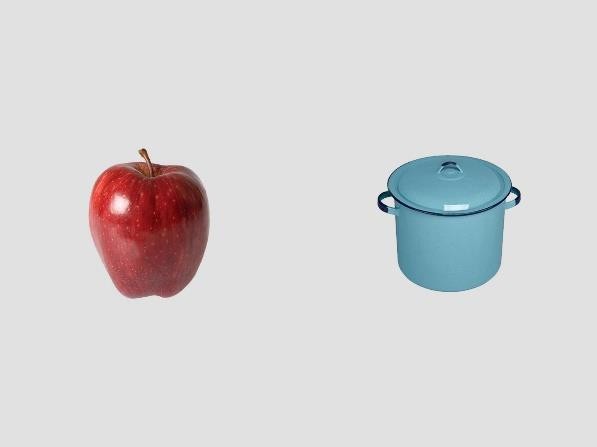 |

| My friend flies the… | 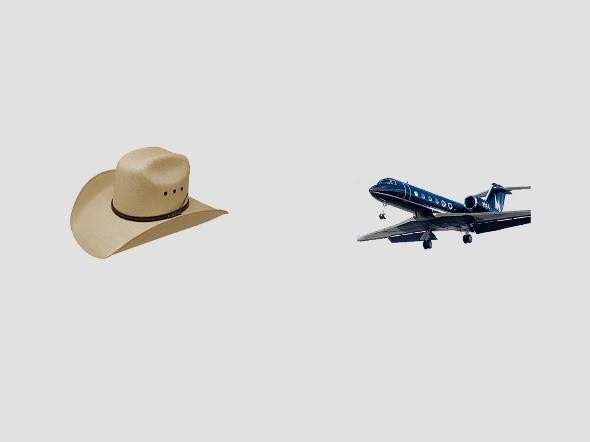 |
| --- | --- |
| The girl plays the… | 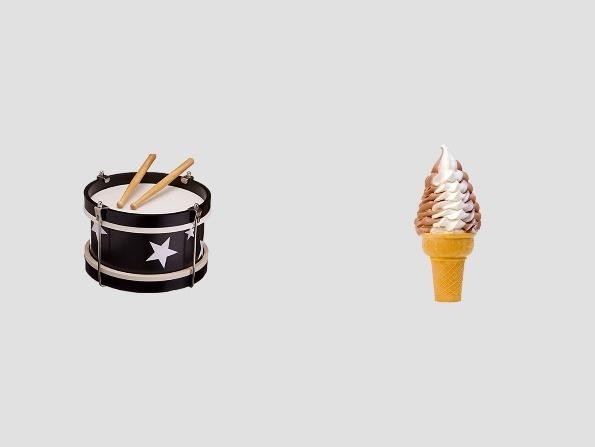 |
| My grandfather kicked the… | 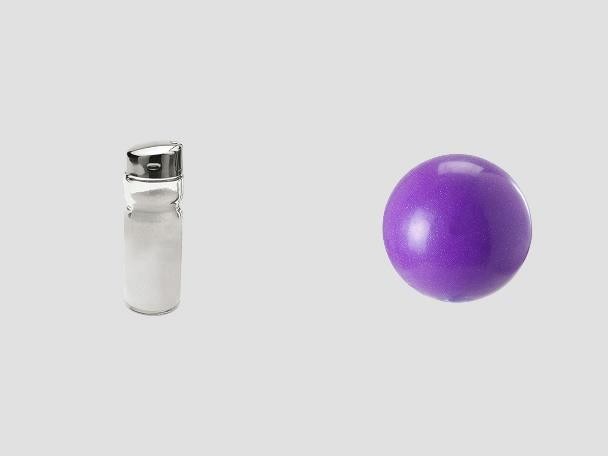 |
| His nephew got on the… | 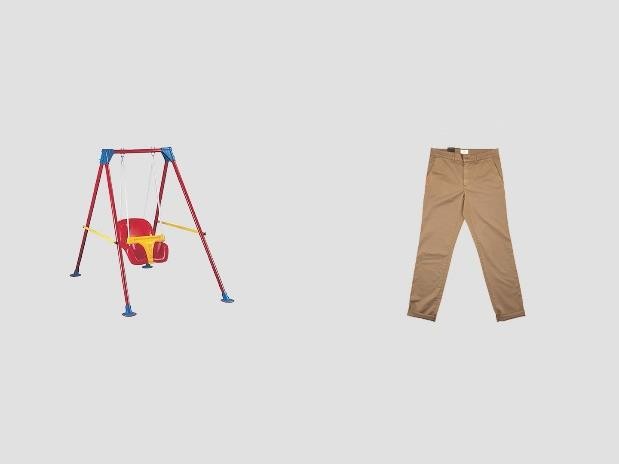 |

| My grandfather washed the… | 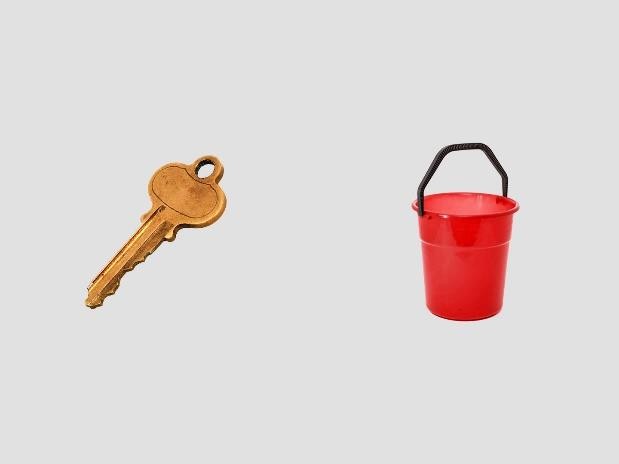 |
| --- | --- |
| My aunt went down the… | 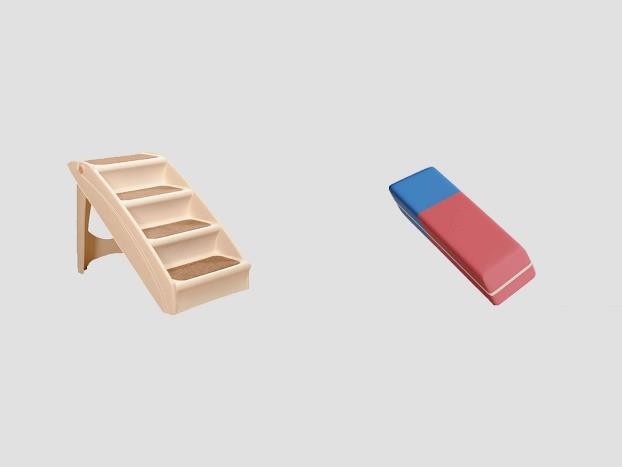 |
| The young woman repaired the… | **…**  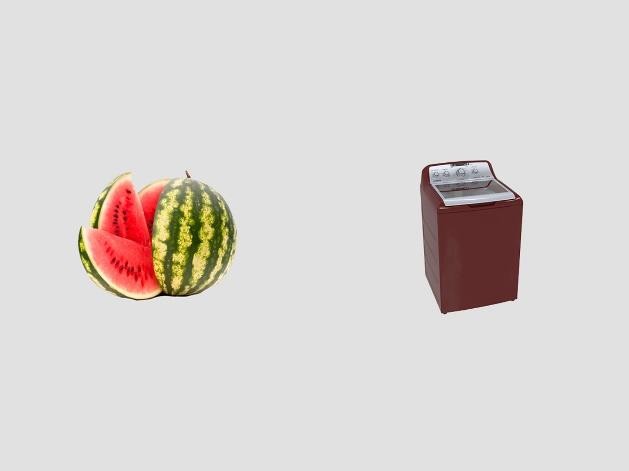 |
| My grandmother mended a… | 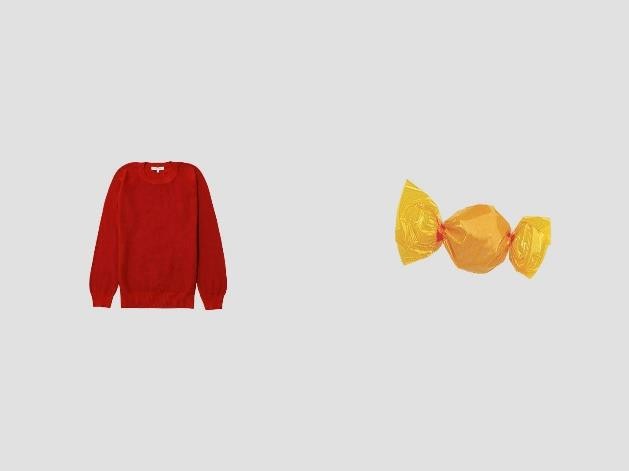 |

| The girl put on the… | 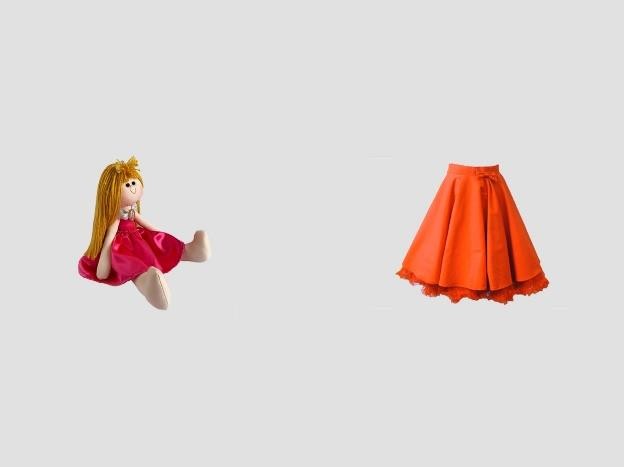 |
| --- | --- |
| The boy plays with a… | 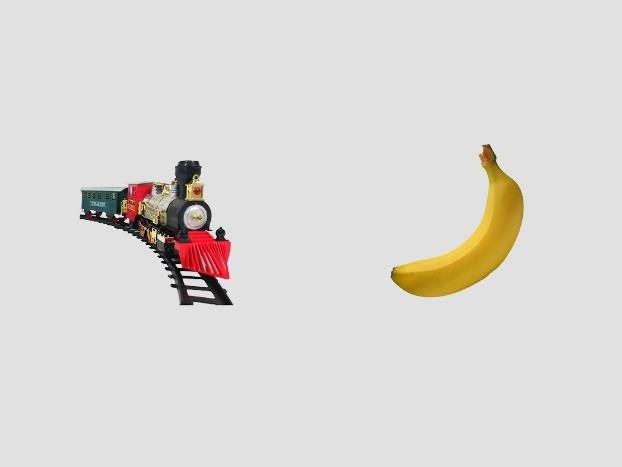 |
| The girl wears a… | 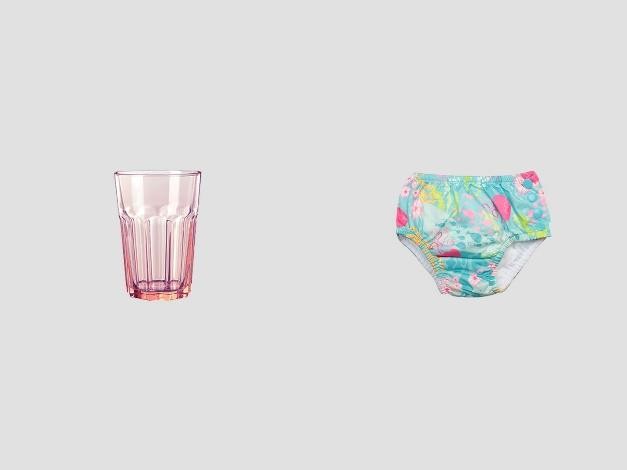 |
| My friend drew in the… | 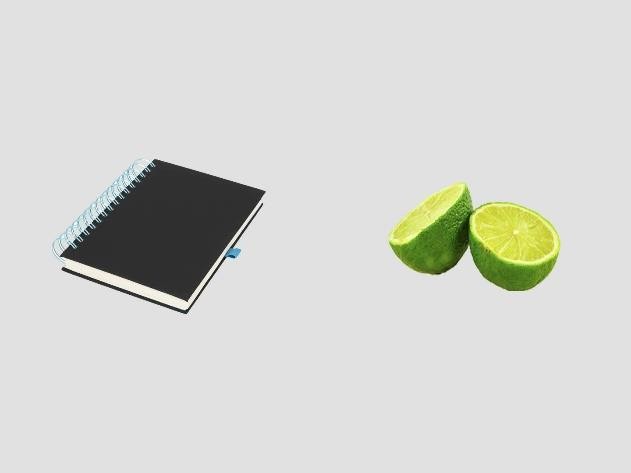 |

| My partner hung up a… | 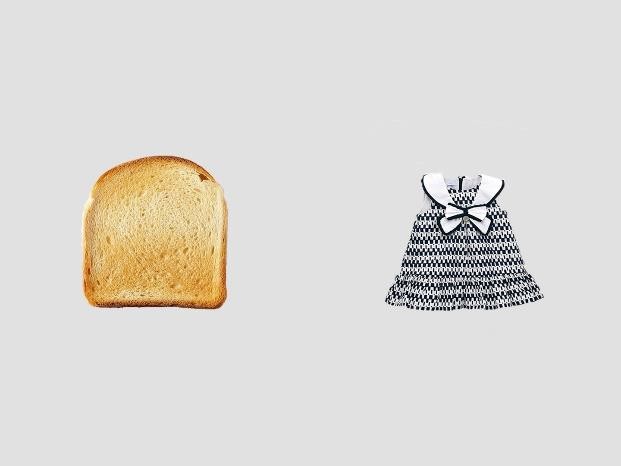 |
| --- | --- |
| My friend heated up a… | 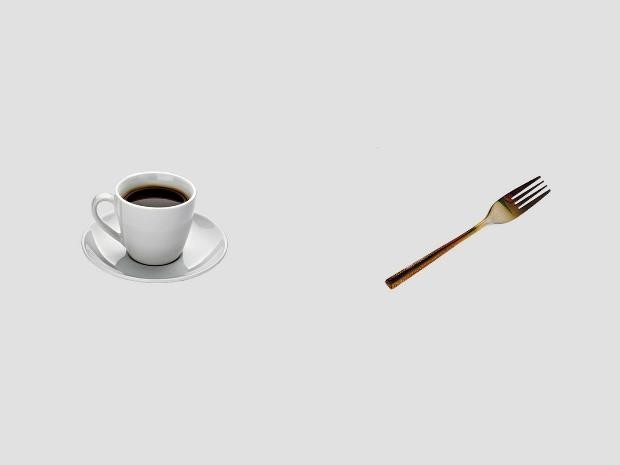 |
| My uncle waited for the… | 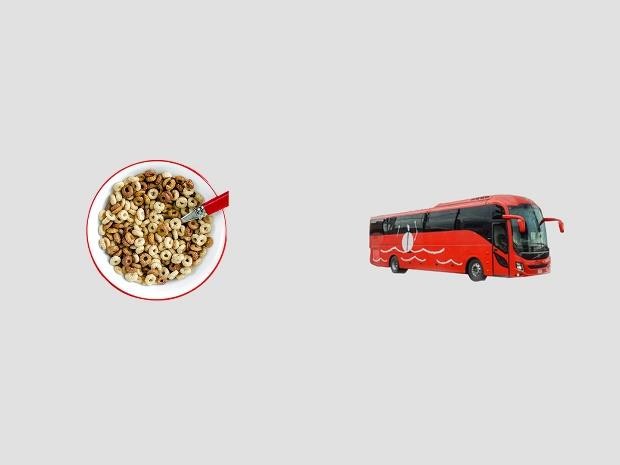 |
| The man dried himself with the… | 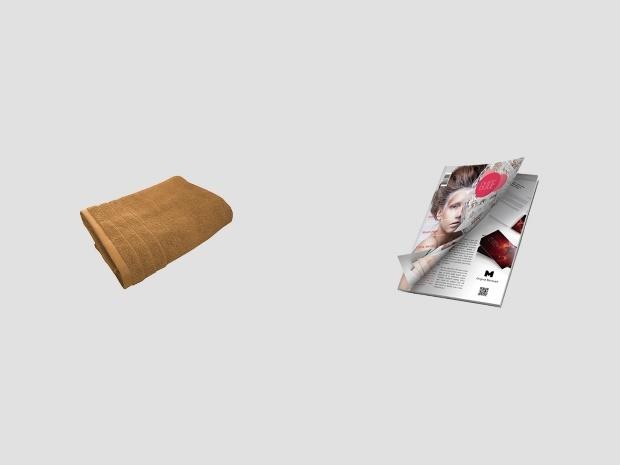 |

| The girl won a… | 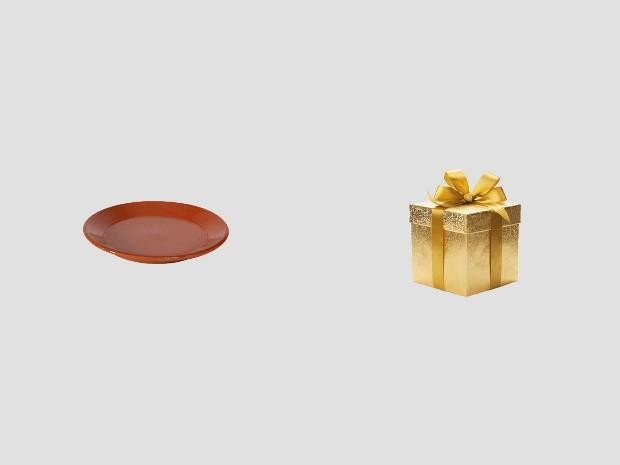 |
| --- | --- |
