# Supplementary material for: Word prediction using closely and moderately related verbs in Down syndrome
Source: Front Psychol. 2022 Oct 3;13:934826. doi: 10.3389/fpsyg.2022.934826 (PMC9574260; doi:10.3389/fpsyg.2022.934826)
Supplement: Supplementary file 2 [file Table_2.pdf]

Supplementary Appendix 1

Closely Related Verb (CV) and Unrelated Verb (UV) Sentences

ID	CV Sentence	UV Sentence	Target	Distractor
1	My father swam in the	My father cleaned the	pool	motorcycle
	Mi papá nadó en la	*Mi papá limpió la*	*alberca*	*motocicleta*
2	In the morning, a . . . barked	In the morning, a . . . arrived	dog	bird
	Por la mañana, ladró un	*Por la mañana, llegó un*	*perro*	*pájaro*
3	The woman read the	The woman lost the	book	sock
	La señora leyó el	*La señora perdió el*	*libro*	*calcetín*
4	My sister swept with a	My sister threw a	broom	spoon
	Mi hermana barrió con una	*Mi hermana tiró una*	*escoba*	*cuchara*
5	My brother wrote with the	My brother hid the	pencil	clock
	Mi hermano escribió con el	*Mi hermano escondió el*	*lápiz*	*reloj*
6	My cousin cut himself with a	My cousin took a	knife	car
	Mi primo se cortó con un	*Mi primo se llevó un*	*cuchillo*	*carro*
7	My aunt ate a	My aunt brought me a	cookie	cup
	Mi tía se comió una	*Mi tía me trajó una*	*galleta*	*taza*
8	My mother talked on the	My mother threw the	telephone	shoe
	Mi mamá habló por el	*Mi mamá aventó el*	*teléfono*	*zapato*
9	My friend fell asleep in the	My friend is in the	bed	chair
	Mi amiga se durmió en la	*Mi amiga está en la*	*cama*	*silla*
10	My partner drank the	My partner opened the	milk	lollipop
	Mi compañera se tomó la	*Mi compañera abrió la*	*leche*	*paleta*
11	My uncle bit a(n)	My uncle paid a(n)	apple	pot
	Mi tió mordió una	*Mi tío pagó una*	*manzana*	*olla*
12	My friend flies the	My friend kept the	airplane	hat
	Mi amigo vuela el	*Mi amigo se quedó con el*	*avión*	*sombrero*
13	The girl plays the	The girl took away my	drum	ice cream
	La niña toca el	*La niña me quitó el*	*tambor*	*helado*
14	My grandfather kicked the	My grandfather took out the	ball	salt
	Mi abuelo pateó la	*Mi abuelo sacó la*	*pelota*	*sal*

Note. Some translations from Spanish to English can change the degree of semantic relationship between the verb and the noun.
