# Supplementary material for: Word prediction using closely and moderately related verbs in Down syndrome
Source: Front Psychol. 2022 Oct 3;13:934826. doi: 10.3389/fpsyg.2022.934826 (PMC9574260; doi:10.3389/fpsyg.2022.934826)
Supplement: Supplementary file 3 [file Table_3.pdf]

Supplementary Appendix 2

Moderately Related Verb (MR) and Unrelated Verb (UV) Sentences

ID	MR Sentence	UV Sentence	Target	Distractor
15	His nephew got on the	His nephew broke the	swing	pants
	Su sobrino se subió al	*Su sobrino rompió el*	*columpio*	*pantalón*
16	My grandfather washed the	My grandfather picked up the	bucket	key
	Mi abuelo lavó la	*Mi abuelo recogió la*	*cubeta*	*llave*
17	My aunt went down the	My aunt looked for the	stairs	eraser
	Mi tía bajó la	*Mi tía buscó la*	*escalera*	*goma*
18	The young woman repaired the	The young woman wants the	washing machine	watermelon
	La joven reparó la	*La joven quiere la*	*lavadora*	*sandía*
19	My grandmother mended a	My grandmother gave us a	sweater	candy
	Mi abuela arregló un	*Mi abuela nos dio un*	*suéter*	*dulce*
20	The girl put on the	The girl kept the	skirt	doll
	La niña se puso la	*La niña guardó la*	*falda*	*muñeca*
21	The boy plays with a	The boy found a	train	banana
	El niño juega con un	*El niño encontró un*	*tren*	*plátano*
22	The girl wears a	The girl needs a	diaper	glass
	La niña usa un	*La niña necesita un*	*pañal*	*vaso*
23	My friend drew in the	My friend bought the	notebook	lemon
	Mi amigo pintó en el	*Mi amigo compró el*	*cuaderno*	*limón*
24	My partner hung up a	My partner had a	dress	bread
	Mi compañera colgó un	*Mi compañera tenía un*	*vestido*	*pan*
25	My friend heated up a	My friend asked for a	coffee	fork
	Mi amiga calentó un	*Mi amiga pidió un*	*café*	*tenedor*
26	My uncle waited for the	My uncle couldn't see the	bus	cereal
	Mi tió esperó el	*Mi tío no veía el*	*autobús*	*cereal*
27	The man dried himself with the	The man adjusted the	towel	magazine
	El señor se secó con la	*El señor acomodó la*	*toalla*	*revista*
28	The girl won a	The girl went for a	gift	dish
	La niña se ganó un	*La niña fue por un*	*regalo*	*plato*
