# Supplementary material for: Word prediction using closely and moderately related verbs in Down syndrome
Source: Front Psychol. 2022 Oct 3;13:934826. doi: 10.3389/fpsyg.2022.934826 (PMC9574260; doi:10.3389/fpsyg.2022.934826)
Supplement: Supplementary file 4 [file Table_4.pdf]

Supplementary Appendix 3
Sentence Plausibility

	CV		UV			MV		UV	
**ID**	**Target**	**Distractor**	**Target**	**Distractor**	**ID**	**Target**	**Distractor**	**Target**	**Distractor**
**1**	92.1	7.46	57.56	47.7	**15**	77	47.66	13.3	7.36
**2**	91.06	7.16	29.86	63.3	**16**	78.2	53.1	54.43	83.2
**3**	92.53	14.1	70.1	58	**17**	84.5	77.26	27.6	46.56
**4**	91.56	9.36	67.46	92.26	**18**	69.43	58.66	9.9	42.33
**5**	89.73	10.1	57.53	11	**19**	76.4	86.2	55.16	72.96
**6**	93	9.26	64.66	92.26	**20**	77.9	63.33	5.96	91.86
**7**	90.43	7.16	84.53	61.53	**21**	87.16	86.06	11.76	74.13
**8**	96.86	10.8	77.16	75.63	**22**	69.4	75.5	53.66	82.6
**9**	90.5	48.93	72.76	71.4	**23**	80.86	64.93	16.66	80.36
**10**	73.43	36.23	66.1	66.1	**24**	94	56.1	13.3	79
**11**	95.56	15.23	71.43	64.86	**25**	57.93	64.5	14.23	80.46
**12**	67.46	42.8	77.73	70.43	**26**	87.4	69.83	18.36	64.66
**13**	94.46	50.7	77.6	72.06	**27**	87.86	70.56	10.76	75.33
**14**	91.03	30.7	68.9	44.2	**28**	88.16	67.7	7.2	76.73

Note. The ID corresponds to the sentences presented in Tables 2 and 3. CV: closely related verb; MV: moderately related verb; UV: unrelated verb.
