# Supplementary material for: Word prediction using closely and moderately related verbs in Down syndrome
Source: Front Psychol. 2022 Oct 3;13:934826. doi: 10.3389/fpsyg.2022.934826 (PMC9574260; doi:10.3389/fpsyg.2022.934826)
Supplement: Supplementary file 5 [file Table_5.pdf]

Supplementary Appendix 4
Association Strength Between Targets and Distractors

	CV		UV			MV		UV	
**ID**	**T**	**D**	**T**	**D**	**ID**	**T**	**D**	**T**	**D**
1	6.451	0	0	0	15	0	0	0	0
2	90.322	0	0	0	16	0	0	0	0
3	87.096	0	0	0	17	12.903	0	0	0
4	80.645	0	0	0	18	0	0	0	0
5	90.322	0	0	0	19	0	0	0	0
6	19.354	0	0	0	20	0	0	0	0
7	6.451	0	0	0	21	0	0	0	0
8	58.064	0	0	0	22	0	0	0	0
9	70.967	0	0	0	23	0	0	0	0
10	0	0	0	0	24	16.129	0	0	0
11	3.225	0	0	0	25	3.225	0	0	0
12	96.774	0	0	0	26	12.903	0	0	0
13	0	0	0	0	27	0	0	0	0
14	64.516	0	0	0	28	0	0	0	0

Note. The ID corresponds to the sentences presented in Tables 2 and 3. CV: closely related verb; MV, moderately related verb; UV: unrelated verb; T, target; D, distractor.
